# Myxovirus resistance protein A to differentiate between viral and non-viral respiratory infections in adults: a prospective study

**DOI:** 10.1080/22221751.2026.2614734

**Published:** 2026-01-16

**Authors:** Mengwei Yan, Nengyong Wang, Liping Yang, Xiaoqi Zhang, Yong Zhang, Weixia Xuan, Xiaoju Zhang, Gang Liu, Herong Wang, Yao Qing, Yeming Wang, Bin Cao

**Affiliations:** a Capital Medical University, National Center for Respiratory Medicine, State Key Laboratory of Respiratory Health and Multimorbidity, National Clinical Research Center for Respiratory Diseases, Institute of Respiratory Medicine, Chinese Academy of Medical Sciences, New Cornerstone Science Laboratory, Department of Pulmonary and Critical Care Medicine, Center of Respiratory Medicine, China–Japan Friendship Hospital, Beijing, People’s Republic of China; b Department of Clinical Laboratory, Guangyuan Central Hospital, Guangyuan, People’s Republic of China; c Department of Respiratory and Critical Care Medicine, Weifang Second People's Hospital, Weifang, People’s Republic of China; d Department of Respiratory and Critical Care Medicine, Henan Provincial People’s Hospital, Zhengzhou, People’s Republic of China; e Department of Clinical Laboratory, Haihe Hospital, Tianjin University, Tianjin, People’s Republic of China; f Department of Respiratory and Critical Care, Haihe Hospital, Tianjin University, Tianjin, People’s Republic of China; g Zybio Inc., Chongqing, People’s Republic of China

**Keywords:** Myxovirus resistance protein A, respiratory infections, viral infection, diagnostics, influenza

## Abstract

Objectives: To assess the diagnostic utility of Myxovirus resistance protein A (MxA) in differentiating between viral and non-viral respiratory infections in adults. Methods: This prospective, multicenter diagnostic accuracy study enrolled adults with acute respiratory infections (ARI) from outpatient and inpatient settings, alongside asymptomatic controls. Peripheral blood was collected for quantitative MxA measurement. Pathogen detection used targeted next-generation sequencing combined with conventional microbiological testing. Aetiological diagnoses were determined using standardized algorithms based on detected pathogens. The diagnostic accuracy of MxA for identifying viral ARIs was calculated. Results: Among 518 ARI patients, 325 had viral pathogens detected, 131 had bacterial/fungal pathogens, and 62 had no pathogen detected. Median MxA levels were significantly higher in viral (123.6 ng/ml; interquartile range [IQR], 56.4–189.6) than bacterial/fungal infections (15.9 ng/ml; IQR, 9.9–38.1; Bonferroni test *p* < 0.001) and controls (n = 158; 8.2 ng/mL; IQR, <5.0–16.4; Bonferroni test *p* < .001). The area under the receiver operating characteristic curve (AUC) for differentiating viral from bacterial/fungal infections was 0.83 (95% confidence interval [CI], 0.79–0.87). At an optimal cutoff of 50 ng/ml, MxA yielded a sensitivity of 77.8% (95% CI, 73.3–82.4%) and a specificity of 80.2% (95% CI, 73.3–87.0%). MxA levels were also elevated in atypical bacterial infections (n = 22; 60.9 ng/mL; IQR, 23.4–114.8), with no significant difference from viral group (Bonferroni test *p* = 0.12). When atypical bacteria were excluded, the AUC for differentiating viral from non-viral infections was 0.80 (95% CI, 0.76–0.84). Conclusions: MxA demonstrates high diagnostic accuracy in distinguishing between viral and non-viral respiratory infections in adults.

## Introduction

Acute respiratory infections (ARI) rank as the most prevalent communicable disease worldwide, imposing a substantial global health burden [1,2]. Viral infections are a leading cause of ARI, accounting for over 35% of cases and exhibiting alternating or concurrent epidemic patterns of distinct respiratory viruses across seasonal periods [3–5]. Diagnostic testing for respiratory viruses other than influenza and SARS-CoV-2, is not routinely incorporated into ARI aetiology screening. Missed or inaccurate diagnosis of viral infections often leads to inappropriate antibiotic use and delays in timely antiviral treatment, potentially worsening clinical outcomes [4,6,7]. Notably, while advancements in pathogen-targeted diagnostic methods, such as viral multiplex polymerase chain reaction (PCR) assays, have improved the detection of viral ARI [8–10], their clinical utility remains limited by high costs, prolonged turnaround times, and sophisticated laboratory requirements, particularly in resource-limited settings. Moreover, the World Health Organization has prioritized the development of rapid, accurate, and affordable biomarkers to identify viral infections [11].

Myxovirus resistance protein A (MxA), an important antiviral protein, shows promise as a host biomarker for differentiating viral from bacterial respiratory infections [12–14]. As a cytoplasmic protein induced by type I and III interferons (IFNs), MxA elevated 1–2 h after infection, peaks at around 16 h [15,16]. It exhibits broad antiviral activity against various respiratory viruses [17,18]. Although several studies have investigated the diagnostic performance of qualitative MxA in combination with C-reactive protein (CRP) testing, as well as quantitative MxA assays in respiratory viral infections [12–14,19], point-of-care testing (POCT) kits for quantitative MxA measurement are still lacking. Furthermore, the diagnostic cutoff value for quantitative MxA in adult patients with respiratory viral infections has yet to be standardized and requires further investigation. Additionally, MxA levels across different viral types, immune states, and disease severities remain insufficiently characterized. These research gaps hinder the broader clinical application of MxA.

In this prospective study, we aimed to evaluate the diagnostic performance of a novel quantitative POCT assay for MxA in respiratory viral infections, establish its optimal threshold for distinguishing between viral and non-viral respiratory infections, and characterize MxA levels across different aetiologies and patient populations.

## Methods

### Study design and participants

This multicenter, diagnostic accuracy study was conducted from December 2023 through July 2024 in outpatients and inpatient settings at five tertiary hospitals in China. Adults (≥ 18 years) presenting with ARI were prospectively enrolled. Patients suspected with upper respiratory tract infections (URTI) were required to exhibit ≥ 2 symptoms/signs: cough, sore throat, nasal congestion, rhinorrhea, hoarseness, or anosmia, with or without fever, and symptom duration of ≤7 days. Lower respiratory tract infections (LRTI) required radiographic evidence of pulmonary infection along with ≥1 of the following: new or worsening respiratory symptoms or signs (cough, phlegm, chest pain, dyspnoea, haemoptysis, moist rales), fever, and peripheral blood leukocytes >10 × 10⁹/L or <4 × 10⁹/L. Exclusion criteria included prior interferon use, major trauma or burns, major surgery, myocardial infarction, stroke, or vaccination, all occurring within the past 30 days. To establish baseline MxA levels in asymptomatic individuals, adults who had not shown any signs of ARI in the past 14 days were recruited from physical examination centres as asymptomatic controls, using the same exclusion criteria as for ARI patients (Supplementary Figure 1). All participants provided written informed consent before sampling. The study was approved by the ethics committees of the participating centres.

### MxA measurements

A venous blood sample was collected from each participant into EDTA tube within 12 h of consent, alongside a respiratory sample from ARI patients. Oropharyngeal swabs (OPs) were collected for URTI, and bronchoalveolar lavage fluid (BALF) or sputum were collected for LRTI at the attending physician's discretion. Whole blood samples were processed within 4 h at site laboratories using an MxA fluorescence immunochromatography kit (Zybio Inc., China) and an automatic fluorescence immunoassay analyzer Q20 (Zybio Inc., China), providing quantitative MxA results within 8 min (details are provided in Appendix 1). Whole blood CRP levels were measured concurrently using the same platform (Zybio Inc., China). The detection limits for MxA were 5 ng/ml (lower limit) and 1000 ng/ml (upper limit). Researchers performing the MxA measurements were blinded to any clinical or pathogen data.

### Pathogen detection and aetiological classification

All respiratory samples, including OPs for URTI and BALF/sputum for LRTI, were tested using a validated multiplex PCR-based targeted next-generation sequencing (tNGS) covering 198 respiratory pathogens (KingCreate, China) (Appendix 2) [20]. In addition, conventional microbiological tests were performed at the discretion of the treating physicians (Appendix 3). The causative pathogens for each ARI patient were determined using established algorithms [3,21–23] (Supplementary Figures 2–3). Patients were classified into the viral-detected group if pathogenic respiratory viruses were identified. Among them, cases with co-detected bacteria, fungi, or mycobacteria were defined as the viral-mixed-detected group [12,24], while those without additional pathogens were categorized as the viral-mono-detected group. Patients negative for respiratory viruses but positive for other pathogens were assigned to the bacterial/fungal-detected group, with further subdivision by pathogen type (typical bacteria, atypical bacteria, mycobacteria, fungi, or mixed pathogens). Patients with no identifiable pathogens were assigned to the no pathogen detected group. The bacterial/fungal-detected group and no pathogen detected group together constituted the non-viral group.

To minimize potential misclassification bias that may arise from relying solely on pathogen detection, which could affect the accuracy of MxA performance evaluation, we reclassified patients into aetiological groups by integrating laboratory findings and detected pathogens with comprehensive clinical diagnoses. A sensitivity analysis was then performed based on this clinically informed classification. Specifically, two experienced respiratory physicians independently reviewed all available clinical data while remaining blinded to both MxA results and the decisions made by their peers. Patients were categorized into viral, non-viral, and inconclusive groups. Assignment to the inconclusive group occurred when no pathogens were detected, or if the detected pathogens did not align with the clinical diagnosis. Final classifications were established by consensus between two independent physicians, with a third independent expert adjudicating in cases of disagreement.

Clinical data were collected from the enrolling center's electronic medical record system through the electronic case report form (eCRF). MxA levels, tNGS results, and all microbiology data from the current visit were recorded. Moreover, a subgroup of patients diagnosed with viral LRTI and exhibiting high baseline MxA levels were invited for follow-up visits, with serial MxA measurements conducted for up to 14 days after enrolment.

### Statistical analyses

MxA levels were analyzed across pathogen groups. The Mann–Whitney U test was used to compare MxA levels between the viral and each non-viral pathogen group, with Bonferroni correction applied for multiple comparisons. Viral cases were further stratified by virus type, including influenza, SARS-CoV-2, adenovirus, respiratory syncytial virus (RSV), rhinovirus, multiple virus co-detections (e.g. influenza-RSV), and other viruses, with differences among viral subgroups assessed using the Kruskal–Wallis test adjusted by Benjamini–Hochberg method.

To evaluate the diagnostic accuracy of MxA for viral ARI, receiver operating characteristic (ROC) curves were generated and the area under the curve (AUC) with 95% confidence intervals (CIs) was calculated. Four primary comparisons were performed: (1) viral-detected vs. non-viral-detected (including both bacterial/fungal and no pathogen-detected groups) groups to evaluate overall diagnostic performance; (2) viral-detected vs. bacterial/fungal-detected group to assess performance in patients with identifiable pathogens; (3) viral-detected vs. bacteria-detected group (including typical and atypical bacteria) to evaluate the ability of MxA to differentiate between viral and bacterial infections; and (4) viral-detected vs. typical bacteria-detected group. For each comparison, the optimal cutoff was determined using the Youden Index, and diagnostic parameters including sensitivity, specificity, positive predictive value (PPV), and negative predictive value (NPV) were calculated. Using the clinically informed classification, we reassessed the diagnostic accuracy of MxA in sensitivity analyses by calculating the AUC and the corresponding diagnostic parameters. ROC analysis was also used to determine the AUC and optimal cutoff for distinguishing viral ARI from asymptomatic controls.

To assess the robustness of MxA-based discrimination across different populations, subgroup analyses were conducted by infection site, immune status, and age, using the optimal cutoff derived from the above models. We also compared MxA levels between febrile and afebrile individuals within 48 h of MxA testing among patients with viral infections. Temporal changes in MxA levels and body temperature were illustrated using line plots for a subgroup of viral LRTI patients who had fever within 48 h of the first MxA testing and underwent serial follow-up visits.

We also constructed a multivariable logistic regression model to evaluate the predictive value of MxA for infection type (viral vs. bacterial/fungal), with adjustment for age, sex, duration of symptoms, comorbidities, immune status, infection site, and fever within 48 h of blood sampling. In addition, an inverse probability of treatment weighting (IPTW) analysis was conducted to reduce imbalance in baseline characteristics between the viral and bacterial/fungal groups. Propensity scores were calculated based on the same covariates included in the logistic regression model and used for weighting. Standardized mean differences (SMD) were calculated for each covariate, and a SMD < 0.1 after weighting indicated adequate balance between groups. A weighted ROC curve was then generated to evaluate the performance of MxA in differentiating viral from bacterial/fungal infections.

Given the elevated MxA levels observed in the atypical bacteria-detected group, we conducted post hoc analyses to assess its diagnostic value for viral ARI, particularly when atypical bacterial infection was suspected or excluded. Specifically, we evaluated its ability to distinguish viral from atypical bacterial ARI, as well as from other non-atypical infections (non-viral-detected group excluding atypical bacteria). For both comparisons, ROC curves were generated, and the AUC values along with optimal cutoff points were calculated. In addition, within the cohort of patients with viral LRTIs, we calculated the proportion requiring different levels of respiratory support across the following MxA level categories: <50, 50–100, 100–200, 200–400, and ≥400 ng/ml.

Additionally, we evaluated the diagnostic performance of CRP in distinguishing between viral and bacterial/fungal-detected groups by constructing ROC curves and calculating AUCs with 95% CIs. The diagnostic accuracy of CRP and MxA in differentiating viral from bacterial/fungal infections was compared using DeLong’s test. On this basis, a multivariable logistic regression model incorporating MxA and CRP was constructed to discriminate viral from bacterial/fungal infections. ROC curves were generated using model-predicted probabilities, and AUCs were calculated. DeLong's test was further applied to compare the diagnostic performance of the combined model with that of CRP alone. A *p*-value of <0.05 was considered significant. All statistical analyses were performed with R version 4.4.1 and SPSS 26.0.

## Results

### Study population

**Figure 1.**
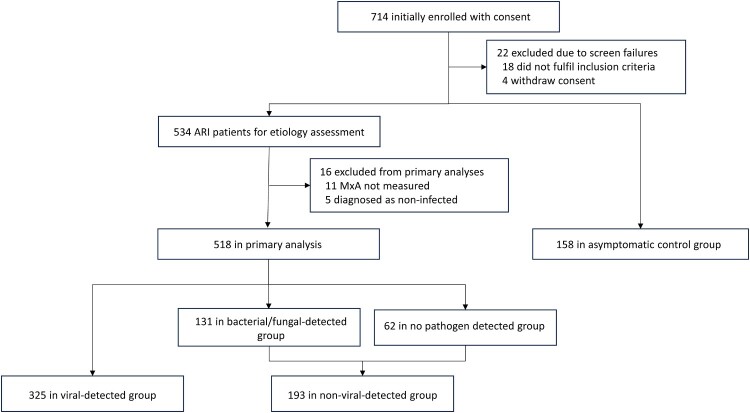
Trial profile. ARI = acute respiratory infection. MxA =  Myxovirus resistance protein A.

A total of 714 patients were enrolled during the study period. After excluding 22 due to screen failures, 534 eligible patients with suspected ARI were assessed for aetiology, and a cohort of 158 asymptomatic controls was included for sub-analysis. Ultimately, 518 ARI patients with available MxA measurements were included in the primary analysis (325 viral-detected, 131 bacterial/fungal-detected, and 62 no pathogen detected) (Figure 1). The median age of the ARI patients was 44 years (interquartile range [IQR], 31–67), with 49 (9.5%) being immunosuppressed. Most patients were recruited from outpatient settings (55.4%), while 144 (27.8%) were from general wards. In total, 300 URTI and 218 LRTI patients were included in the study, with detailed baseline characteristics for each group provided in Supplementary Table 1 and 2.

### MxA levels among different pathogen groups

**Figure 2.**
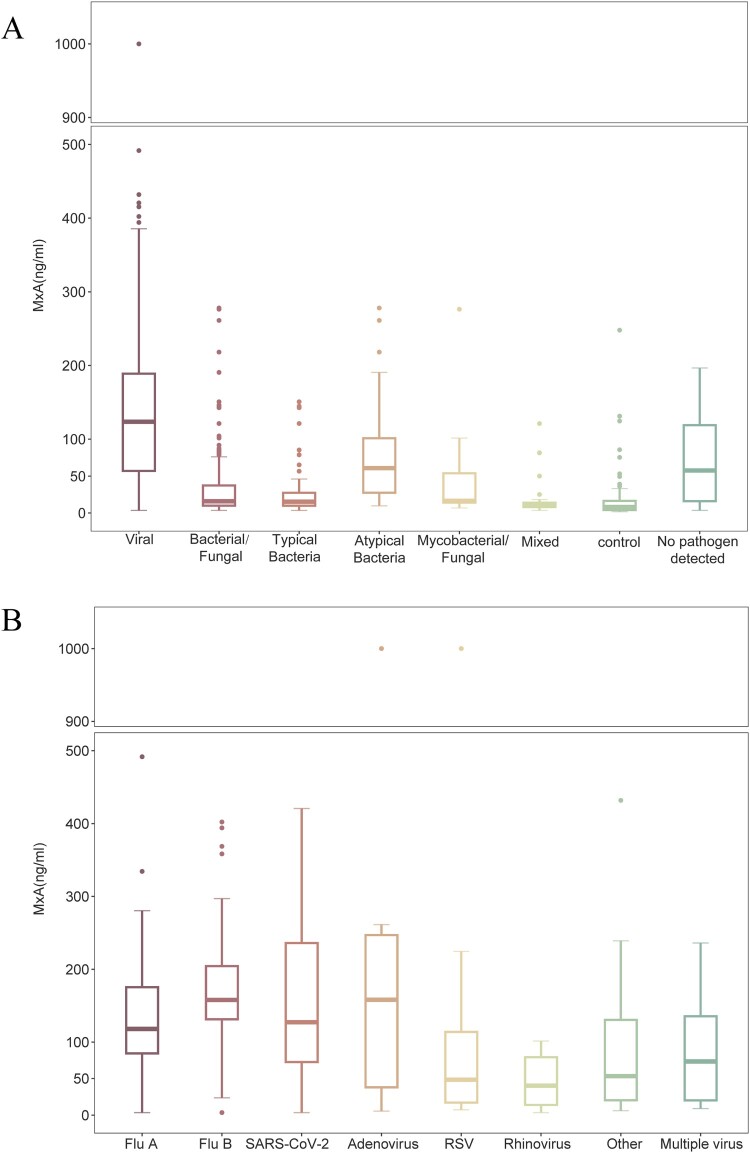
Boxplots of MxA levels in ARI patients and asymptomatic controls. (A) Boxplots of MxA levels in ARI patients and asymptomatic controls, categorized by pathogen group. (B) Boxplots of MxA levels in ARI patients with viral infections, stratified by virus subgroups. Boxes indicate median and interquartile range, with whiskers indicate 1.5 times of IQR, and dots marking outliers beyond the whiskers. MxA = Myxovirus resistance protein A. ARI = acute respiratory infection. Flu A = influenza A. Flu B = influenza B. RSV = respiratory syncytial virus.

The median MxA level in viral-detected group was 123.6 ng/ml (IQR, 56.4–189.6 ng/ml), significantly higher than bacterial/fungal-detected group (15.9 ng/ml; IQR, 9.9–38.1 ng/ml; *p* < 0.001, Bonferroni test *p* < 0.001), typical bacteria-detected group (15.3 ng/ml; IQR, 9.8–28.4 ng/ml; *p* < 0.001, Bonferroni test *p* < 0.001), asymptomatic controls (8.2 ng/ml; IQR, <5.0–16.4 ng/ml; *p* < 0.001, Bonferroni test *p* < 0.001), and no pathogen detected group (57.7 ng/ml; IQR, 15.7–120.6 ng/ml; *p* < 0.001, Bonferroni-adjusted *p* < 0.001). Notably, atypical bacteria-detected group exhibited elevated MxA levels (median 60.9 ng/mL; IQR, 23.4–114.8 ng/mL; *p* = 0.02, Bonferroni test *p* = 0.12) (Figure 2A and Supplementary Table 3).

Among viral infections, MxA levels were significantly higher in viral-mono-detected (136.3 ng/ml; IQR, 85.7–193.5 ng/ml) than viral-mixed-detected group (69.0 ng/ml; IQR, 18.7–167.1 ng/ml; *p* < 0.001) (Supplementary Figure 4 and Supplementary Table 3). Patients with adenovirus exhibited the highest MxA levels (median 158.2 ng/mL; IQR, 27.1–446.0 ng/mL), while patients with RSV and rhinovirus had lower MxA levels (median 48.4 ng/ml; IQR, 16.4–120.0 ng/ml; median 40.2 ng/ml; IQR, 11.7–89.4 ng/ml, respectively) (Figure 2B, Supplementary Table 3 and 4).

### Diagnostic performance of MxA for viral ARI

**Figure 3.**
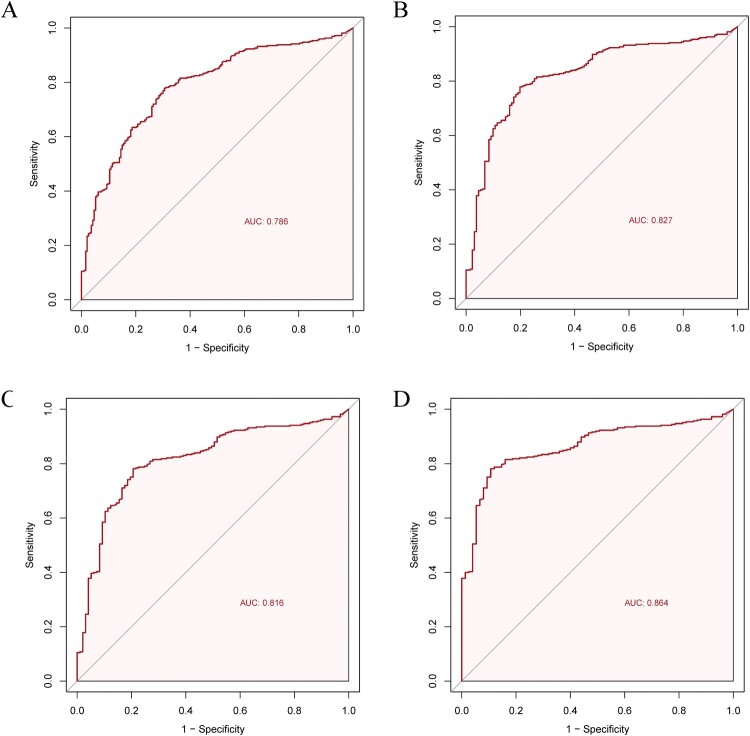
Receiver operating characteristic curves of MxA to discriminate respiratory viral infections. (A) ROC curve for MxA to discriminate viral from non-viral ARI (including both bacterial/fungal-detected and no pathogen detected groups). (B) ROC curve for MxA to discriminate viral from bacterial/fungal ARI (patients with any non-viral pathogen detected). (C) ROC curve for MxA to discriminate viral from bacterial ARI (including both typical and atypical bacteria). (D) ROC curve for MxA to discriminate viral from atypical bacterial ARI. MxA = Myxovirus resistance protein A. ARI = acute respiratory infection. ROC = receiver operating characteristic.

Overall, MxA distinguished viral from non-viral ARI with an AUC of 0.79 (95% CI, 0.75–0.83). When the analysis was restricted to patients with identifiable pathogens, the AUC increased to 0.83 (95% CI, 0.79–0.87) for distinguishing viral from bacterial/fungal infections. At an optimal cutoff of 50 ng/ml, MxA demonstrated a sensitivity of 77.8% (95% CI, 73.3–82.4) and a specificity of 80.2% (95% CI, 73.3–87.0). The highest diagnostic performance was observed when differentiating viral from typical bacterial infections, with an AUC of 0.86 (95% CI, 0.83–0.90) and a specificity of 89.3% (95% CI, 82.3–96.3) (Table 1 and Figure 3). In distinguishing viral ARI from asymptomatic controls, MxA showed excellent performance, with an AUC of 0.93 (95% CI, 0.90–0.95) and an optimal cutoff of 36 ng/ml (Supplemental Figure 5).

**Table 1.** Diagnostic accuracy of MxA for identifying viral infections in ARI patients

	Optimal Cutoff (ng/ml)	AUC (95% CI)	Sensitivity % (95% CI)	Specificity % (95% CI)	PPV % (95% CI)	NPV % (95% CI)
*All ARI patients (including those with no pathogen detected)*						
Viral vs non-viral	50	0.79 (0.75, 0.83)	77.8 (73.3, 82.4)	69.4 (62.9, 75.9)	81.1 (76.7, 85.4)	65.0 (58.5, 71.6)
*ARI patients with pathogens detected*						
Viral vs bacterial/fungal	50	0.83 (0.79, 0.87)	77.8 (73.3, 82.4)	80.2 (73.3, 87.0)	90.7 (87.3, 94.1)	59.3 (52.1, 66.6)
Viral vs bacterial	47	0.82 (0.77, 0.86)	78.2 (73.7, 82.6)	79.4 (71.3, 87.4)	92.7 (89.6, 95.8)	52.0 (44.0, 60.1)
Viral vs typical bacterial	47	0.86 (0.83, 0.90)	78.2 (73.7, 82.6)	89.3 (82.3, 96.3)	96.9 (94.9, 99.0)	48.6 (40.2, 56.9)

Data represent optimal cutoffs and corresponding diagnostic accuracy measures (95% CI) derived from receiver operating characteristic analyses in four comparisons: (1) viral-detected group vs. non-viral-detected group (including both bacterial/fungal-detected and no pathogen detected groups); (2) viral-detected group vs. bacterial/fungal-detected group (patients with any non-viral pathogen detected); (3) viral-detected group vs. bacteria-detected group (including both typical and atypical bacteria); (4) viral-detected group vs. typical bacteria-detected group.

ARI: acute respiratory tract infection; MxA: Myxovirus resistance protein A; CI: confidence interval; AUC: area under the curve.

### Diagnostic performance of MxA for viral ARI across diverse populations

The diagnostic accuracy of MxA in distinguishing between viral and bacterial/fungal ARI varies across different subpopulations. Using a threshold of 50 ng/ml, the specificity remained relatively consistent across infection sites, immune status, and age groups (Table 2). However, sensitivity notably declined in patients with LRTIs (48.5%; 95% CI, 38.9–58.2) and in immunosuppressed individuals (33.3%; 95% CI, 19.8–50.4). In viral LRTI patients, MxA levels were significantly higher in those with fever (median 61.5 ng/mL; IQR, 18.1–199.0 ng/ml) than in those without fever at sampling (22.6 ng/mL; IQR, 9.9–58.1 ng/ml; *p* = 0.011) (Supplementary Figure 6). Longitudinal assessment of 9 febrile viral LRTI patients revealed a consistent decline in MxA levels, paralleling the resolution of fever (Supplementary Figure 7). Excluding afebrile viral cases improved the diagnostic accuracy for LRTI, increasing the AUC from 0.68 (95% CI, 0.61–0.76) to 0.74 (95% CI, 0.65–0.82) (Table 2).

**Table 2.** Diagnosis accuracy of MxA in differentiating between viral and bacterial/fungal ARI across subgroups.

Subgroup Analysis	Number of patients classified as viral (%)	AUC (95% CI)	Cutoff (ng/ml)	Sensitivity % (95% CI)	Specificity % (95% CI)	PPV % (95% CI)	NPV % (95% CI)
*Site*							
URTI	226/257(87.9%)	0.87 (0.78, 0.94)	50	90.7 (86.2, 93.8)	71.0 (53.4, 83.9)	95.8 (92.2, 97.8)	51.2 (36.8, 65.4)
LRTI	99/199 (49.5%)	0.68 (0.61, 0.76)	50	48.5 (38.9, 58.2)	82.0 (73.3, 88.3)	72.7 (61.0, 82.0)	61.7 (53.2, 69.5)
LRTI- Immunocompetent ^a^	71/176 (40.3%)	0.73 (0.65-0.81)	50	56.3 (44.8, 67.3)	82.2 (73.1, 88.8)	71.4 (58.5, 81.6)	70.5 (61.2, 78.4)
LRTI-fever ^b^	63/163 (38.7%)	0.74 (0.65, 0.82)	50	57.1 (44.9, 68.6)	82.0 (73.3, 88.3)	66.7 (53.4, 77.8)	75.2 (66.4, 82.4)
*Immunity*							
Immunosuppressed	33/43 (76.7%)	0.63 (0.42, 0.86)	50	33.3 (19.8, 50.4)	80.0 (49.0, 94.3)	84.6 (57.8, 95.7)	26.7 (14.2, 44.4)
Immunocompetent	292/413 (70.7%)	0.86 (0.82, 0.90)	50	82.9 (78.1, 86.8)	79.3 (71.3, 85.6)	90.6 (86.5, 93.6)	65.8 (57.7, 73.0)
*Age (y)*							
< 65	256/328 (78.0%)	0.84 (0.78, 0.89)	50	84.4 (79.4, 88.3)	72.2 (61.0, 81.2)	91.5 (87.2, 94.4)	56.5 (46.3, 66.2)
≥ 65	69/128 (53.9%)	0.74 (0.65, 0.83)	50	53.6 (42.0, 64.9)	88.1 (77.5, 94.1)	84.1 (70.6, 92.1)	61.9 (51.2, 71.6)

Data represent subgroup diagnostic accuracy measures (95% CI), calculated using the 50 ng/ml threshold derived from the overall viral vs. non-viral ARI model.

^a^ LRTI-Immunocompetent subgroup includes patients with lower respiratory tract infections (LRTIs) who were immunocompetent (i.e. without immunosuppression).

^b^ LRTI-Fever subgroup includes LRTI patients excluding those with viral infections who were afebrile within 48 h of MxA testing.

MxA: Myxovirus resistance protein A; ARI: acute respiratory infection; URTI: upper respiratory infections; LRTI: lower respiratory infections; CI: confidence interval; AUC: area under the curve; NPV: negative predictive value; PPV: positive predictive value.

For URTIs, due to the controversial opinions regarding the pathogenic relevance of bacteria detected [23], we evaluated MxA in patients likely requiring intervention (excluding typical bacterial infections with WBC <10 × 10⁹/L). MxA showed good diagnostic accuracy in this subgroup, with an AUC of 0.81 (95% CI, 0.65–0.96) (Supplementary Figure 8).

### Sensitivity analyses

Based on the clinically informed aetiological classification, 321 patients were categorized into the viral group, 106 into the bacterial/fungal group, and 91 were classified as having an inconclusive aetiology. The median MxA level in the viral group was 118.4 ng/mL (IQR, 54.2–189.3 ng/mL), which was significantly higher than that in the bacterial/fungal group (15.4 ng/mL; IQR, 9.8–37.0 ng/mL; *p* < 0.001) and the typical bacterial group (14.6 ng/mL; IQR, 9.7–26.0 ng/mL; *p* < 0.001). No significant difference was observed compared with the atypical bacterial group (82.2 ng/mL; IQR, 28.9–133.8 ng/mL; *p* = 0.053) (Supplementary Figure 9). The diagnostic performance of MxA for identifying viral ARI based on the clinically informed classification was largely consistent with that derived from the pathogen-based aetiological classification (Supplementary Table 5). Specifically, MxA demonstrated an AUC of 0.83 (95% CI, 0.78–0.87) in differentiating viral from bacterial/fungal ARIs, with an optimal cutoff value of 51 ng/mL, yielding a sensitivity of 77.3% (95% CI, 72.7–81.8) and a specificity of 81.1% (95% CI, 73.7–88.6). Similarly, MxA discriminated viral from bacterial infections with an AUC of 0.82 (95% CI, 0.77–0.87) and achieved an AUC of 0.88 (95% CI, 0.84–0.92) when distinguishing viral from the typical bacterial group.

After adjustment for potential confounders, the multivariable logistic regression model demonstrated that MxA remained an independent predictor of infection type (viral vs. bacterial/fungal), with an adjusted odds ratio of 1.02 per ng/mL (95% CI, 1.01–1.03; *p* < 0.001; Supplementary Table 6). Consistently, the IPTW weighted ROC analysis yielded an AUC of 0.83 (95% CI, 0.79–0.87) for differentiating viral from bacterial/fungal infections (Supplementary Figure 10).

### Post hoc analyses

When atypical bacterial infections were not considered in the differential diagnosis, MxA yielded an AUC of 0.80 (95% CI, 0.76–0.84) for distinguishing viral from non-viral-detected group (excluding atypical bacteria), with the optimal cutoff remaining at 50 ng/ml. When distinguishing viral from atypical bacterial infections, the AUC was 0.65 (95% CI, 0.53–0.77), and the optimal diagnostic threshold increased to 105 ng/ml (Supplementary Table 7). Among patients with viral LRTIs whose MxA levels exceeded 50 ng/ml, the proportion requiring mechanical ventilation increased progressively with rising MxA levels (Supplementary Figure 11).

### Diagnostic performance of CRP and CRP–MxA models for differentiating viral and bacterial/fungal ARI

The AUC of CRP for discriminating viral from bacterial/fungal infections was 0.64 (95% CI, 0.58–0.69; Supplementary Figure 12A). At an optimal cutoff of 7 mg/L, CRP showed a sensitivity of 80.2% (95% CI, 73.3–87.0) and a specificity of 46.5% (95% CI, 41.0–51.9). Its diagnostic performance was significantly inferior to that of MxA (*p* < 0.001). The combined CRP–MxA model yielded an AUC of 0.82 (95% CI, 0.78–0.86; Supplementary Figure 12B), which was significantly higher than that of CRP alone (*p* < 0.001).

## Discussion

In this multicenter prospective study, we quantitatively measured blood MxA levels using fluorescent immunochromatography in adults with ARIs. Our findings demonstrate that MxA could accurately differentiate between respiratory viral and non-viral infections. With a cutoff value of 50 ng/ml, MxA demonstrated optimal diagnostic performance in identifying respiratory viral infections.

This study enrolled a comprehensive cohort of adult ARI patients across outpatient, inpatient, and ICU settings, encompassing a broad spectrum of real-world scenarios, which is essential for identifying appropriate clinical scenarios and target populations for MxA testing. Consistent with previous research, our findings confirm that MxA demonstrates strong diagnostic performance for viral URTIs [12,25,26], supporting its potential as a rapid screening tool in outpatient settings. While the diagnostic sensitivity of MxA for viral LRTIs was relatively low, excluding viral cases without fever markedly enhanced its performance. Since fever typically indicates the acute phase of infection, whereas afebrile status may reflect recovery, and given that MxA levels were observed to decrease alongside fever resolution in febrile patients, the significantly higher MxA levels observed in febrile patients suggest that MxA may decline with clinical improvement. Additionally, some respiratory viruses may evade the host immune response by inhibiting the interferon signalling pathway [27–30], leading to lower MxA levels. These findings indicate that for LRTI patients, comprehensive and appropriate microbiological testing is essential. MxA is better suited as an adjunctive tool for distinguishing viral infections, rather than being relied upon as the sole method for definitive aetiological diagnosis in LRTI patients. Moreover, the dynamic changes in MxA levels may help inform decisions regarding the cessation or initiation of antiviral treatment. Future studies should further validate the clinical utility of MxA in guiding antiviral treatment and explore the association between MxA defects and clinical outcomes.

MxA demonstrates limited ability to differentiate viral from atypical bacterial respiratory infections. Since MxA production is regulated by interferons, other factors that activate interferon pathways, including intracellular bacterial infections, may also elevate MxA levels [31,32]. Consistent with our hypothesis, we observed elevated MxA levels in patients with atypical bacterial infections, which are primarily caused by intracellular pathogens. This finding is consistent with the results reported by Rhedin et al. and highlights the importance of cautious interpretation of elevated MxA levels [13]. Subsequent post hoc analyses further supported this observation, suggesting that a higher cutoff value may be necessary to improve specificity when atypical bacterial infections cannot be ruled out. Therefore, during non-viral epidemic periods or when clinical features cannot definitively rule out atypical bacterial respiratory infections, relying on elevated MxA levels alone may introduce diagnostic uncertainty. We recommend complementing MxA measurements with microbiological testing to support clinical decision-making. Furthermore, large-scale studies are needed to better characterize MxA profiles in patients with atypical bacterial infections and to further evaluate its diagnostic performance in distinguishing viral from atypical bacterial respiratory infections.

Contrary to previous studies [13,33], we observed that a proportion of patients infected with RSV, rhinovirus, human metapneumovirus (HMPV), or human coronaviruses did not exhibit elevated MxA levels. Different respiratory viruses vary significantly in their capacity to induce a systemic type I/III interferon response, which is the primary driver of MxA production. For instance, compared to influenza virus and HMPV, RSV is known to elicit a milder and more delayed interferon response [34,35], which could translate to lower or less consistent MxA elevation. Furthermore, current evidence suggests that MxA increases in response to pathogenic viral infections, whereas commensal viruses, which do not trigger a robust host immune response, fail to induce MxA production [36]. Supporting this notion, individuals with these viral detections in our cohort had a relatively low proportion of fever. Fever not only indicates the acute phase of infection but also reflects a more pronounced systemic immune response, which has been associated with higher MxA levels [33]. Taken together, the variability in MxA levels among these viral infections may be attribute to their mild pathogenicity or to the possibility that these viruses act as bystanders in mixed infections [13], indicating that MxA levels may assist clinicians in identifying whether a detected virus is the primary contributor to the current ARI.

The optimal threshold for distinguishing viral from non-viral respiratory infections using MxA measured by fluorescence immunochromatography was 50 ng/mL in this study, which is lower than values reported in earlier research [13,19,37,38]. Previous studies have primarily focused on paediatric populations. For instance, a prospective study in children aged 1–59 months with LRTIs reported an optimal threshold of 430 ng/mL using enzyme immunoassay [13]. Similarly, another study using sandwich immunoassay in children aged 0–16 years identified a threshold of 200 ng/mL for distinguishing viral infections from uninfected controls [37]. This discrepancy may be attributed to the differing baseline MxA levels between children and adults, with the median baseline MxA level in uninfected children being 110 µg/L, significantly higher than in adults (approximately 10 μg/L) [33]. Furthermore, this study included a broader patient population, and MxA levels varied according to fever status and disease severity, which may also account for the different threshold identified here. Further validation in more defined subpopulations is warranted. Notably, consistent with prior observations in COVID-19 patients [39], we found that MxA levels were associated with disease severity, with higher MxA levels potentially indicating more severe disease, as reflected by the need for greater respiratory support. This suggests that MxA could serve not only as a diagnostic biomarker but also as a prognostic tool.

The diagnostic performance of MxA for respiratory viral infections in immunosuppressed populations remains controversial. While some studies suggest that MxA maintains acceptable diagnostic accuracy in immunocompromised patients [38,40], others reported impaired MxA production [41]. We propose that immunocompetent patients in the acute phase of infection represent the optimal population for maximizing the diagnostic utility of MxA in distinguishing respiratory viral infections, whereas relying solely on MxA testing to exclude viral infections in immunosuppressed ARI patients is not advisable due to its limited sensitivity. However, given its relatively high specificity, MxA could be valuable for monitoring infection resolution in patients with confirmed viral infections who exhibit elevated MxA levels. Large, well-powered studies in immunocompromised patients with respiratory infections are needed to confirm these findings.

Despite advances in pathogen detection technologies such as tNGS, mNGS, and multiplex PCR, identifying the causative pathogens in all respiratory infection cases remains challenging due to inherent technical limitations [20,42,43]. Consequently, clinicians still face diagnostic uncertainty when microbiological tests are negative, relying heavily on clinical judgment for management decisions. Against this backdrop, our study, employing a comprehensive diagnostic strategy combining tNGS with conventional microbiological testing, revealed that patients with no pathogen detected exhibited relatively low MxA levels. This observation highlights a key potential utility of MxA, that in diagnostically uncertain cases, a low or negative MxA result may provide objective evidence against an active viral infection, thereby increasing confidence in considering non-viral or non-infectious aetiologies and supporting more targeted clinical decision-making.

This study has several limitations that should be acknowledged. First, patients in the bacterial/fungal group were generally older, had a higher proportion of LRTI, and exhibited a longer median duration of symptoms compared to those with viral infections. These imbalances were likely influenced by the inherent aetiological patterns of ARI. Notably, although MxA showed reduced sensitivity for diagnosing viral infections in LRTI patients, its diagnostic utility may have been underestimated, as many of these patients had longer symptom durations prior to enrolment and were possibly in the recovery phase. Third, in the absence of a gold standard for determining the aetiology of ARI, we adopted a pathogen-based classification algorithm to enhance objectivity. However, it should be noted that some patients may be incorrectly assigned to the wrong pathogen group, which could lead to an underestimation of MxA's diagnostic performance. To minimize potential bias from relying solely on detected pathogens, we also performed a sensitivity analysis that incorporated both detected pathogens and clinical diagnosis for aetiological classification. What's more, given the inherent limitations of pathogen detection technologies, some patients in the no pathogen detected group may have true viral infections that were missed because of low viral load or infection with viruses not included in the testing panel. Consequently, the diagnostic specificity of MxA may be underestimated in analyses evaluating its performance in distinguishing viral from the non-viral group, which comprised both bacterial/fungal-detected and no-pathogen-detected patients. Fourth, as pathogen testing was not performed in the asymptomatic control group, the potential presence of asymptomatic carriers could not be identified.

Our findings indicate that MxA is a reliable biomarker for diagnosing viral ARIs, with particularly strong diagnostic performance in fever clinic and outpatient settings. It may also serve as an adjunctive diagnostic tool for adult patients with LRTI. Moreover, MxA levels appear to reflect the disease stage of viral ARIs. Future research should further explore the role of MxA in guiding antiviral treatment decisions and its potential prognostic value in viral ARIs.

## Supplementary Material

Supplemental Material

Appendix2.xlsx

Appendix1.xlsx

MxA_Manuscript_revised_clean_nolegend.docx

Appendix3.docx

## Data Availability

Qualified researchers may apply for individual patient-level data by contacting the corresponding authors upon reasonable request.

## Appendices

### Appendix 1. MxA quantitative measurement assay

Whole blood samples were collected for MxA quantitative measurement within 4 h of sampling using a fluorescence immunochromatography (Zybio Inc., Chongqing, China) MxA detection kit and the Q20 automatic fluorescence immunoassay analyzer (Zybio Inc., Chongqing, China).

The MxA fluorescence immunochromatography detection strip consists of a sample pad, a conjugate pad, a nitrocellulose membrane, an absorbent pad, and a PVC backing plate. The conjugate pad is pre-coated with fluorescent microsphere-labelled anti-human MxA mouse monoclonal IgG1 (clone 22D4), as well as a fluorescent microsphere-labelled dinitrophenol (DNP)-bovine serum albumin (BSA). The test line on the nitrocellulose membrane is coated with anti-human MxA mouse monoclonal IgG1 (clones 27H9 and 19A8), while the control line is coated with anti-DNP antibodies.

Following the standard operating procedure, the whole blood sample is placed on the sample rack of the Q20 analyzer, which automatically aspirates 5 μl of whole blood and mixes it with the MxA sample diluent (Zybio Inc., Chongqing, China). A 70 μl aliquot of the mixture is then added to the sample pad of the test strip. The mixture migrates along the strip, where MxA in the mixture binds to the fluorescently labelled antibodies on the conjugate pad and is subsequently captured by the immobilized antibodies on the test line, generating a fluorescent signal proportional to the MxA level. Regardless of the MxA levels, the anti-DNP antibodies on the control line bind to the fluorescently labelled DNP-BSA complex, generating a constant fluorescent signal.

The Q20 analyzer automatically measures the fluorescence intensities of the test line and control line and calculates the MxA level based on a manufacturer-predefined internal standard curve. The total testing time is 8 min.

### Appendix 2. Pathogen panel (198 pathogens) for targeted next-generation sequencing (tNGS)

**Table:**

Pathogen types	Pathogens
DNA viruses	BK polyomavirus
DNA viruses	Human adenovirus 1
DNA viruses	Human adenovirus 2
DNA viruses	Human adenovirus 21
DNA viruses	Human adenovirus 5
DNA viruses	Human adenovirus 55
DNA viruses	Human adenovirus 57
DNA viruses	Human adenovirus 6
DNA viruses	Human adenovirus 7
DNA viruses	Human adenovirus B3
DNA viruses	Human adenovirus E4
DNA viruses	Herpes simplex virus 1
DNA viruses	Herpes simplex virus 2
DNA viruses	Varicella Zoster Virus
DNA viruses	Cytomegalo Virus
DNA viruses	Human herpes virus-6
DNA viruses	Human herpes virus-7
DNA viruses	Human bocavirus 1
DNA viruses	Human bocavirus 2
DNA viruses	Human bocavirus 3
DNA viruses	Human bocavirus 4
DNA viruses	Epstein-Barr virus
DNA viruses	Human mastadenovirus
DNA viruses	Human mastadenovirus B
DNA viruses	Human mastadenovirus C
DNA viruses	Human mastadenovirus D
DNA viruses	Human parvovirus B19
DNA viruses	Human adenovirus 11
DNA viruses	Human adenovirus 14
DNA viruses	Human adenovirus 34
DNA viruses	Human adenovirus 35
DNA viruses	Human herpes virus-6A
DNA viruses	Human herpes virus-6B
DNA viruses	JC polyomavirus
DNA viruses	WU Polyomavirus
RNA viruses	Coxsackievirus A10
RNA viruses	Coxsackievirus A16
RNA viruses	Coxsackievirus A2
RNA viruses	Coxsackievirus A5
RNA viruses	Coxsackievirus A6
RNA viruses	Coxsackievirus B3
RNA viruses	Echovirus E18
RNA viruses	Echovirus E30
RNA viruses	Enterovirus
RNA viruses	Enterovirus A
RNA viruses	Enterovirus A71
RNA viruses	Enterovirus B
RNA viruses	Enterovirus C
RNA viruses	Enterovirus D
RNA viruses	Enterovirus D68
RNA viruses	Human coronavirus 229E
RNA viruses	Human coronavirus HKU1
RNA viruses	Human coronavirus NL63
RNA viruses	Human coronavirus OC43
RNA viruses	Human metapneumovirus
RNA viruses	Human orthorubulavirus 2
RNA viruses	Human orthorubulavirus 4
RNA viruses	Human respiratory syncytial virus A
RNA viruses	Human respiratory syncytial virus B
RNA viruses	Human respirovirus 1
RNA viruses	Human respirovirus 3
RNA viruses	Influenza A virus
RNA viruses	Influenza A(H1N1) virus
RNA viruses	Influenza A(H3N2) virus
RNA viruses	Influenza A(H5N1) virus
RNA viruses	Influenza A(H7N9) virus
RNA viruses	Influenza B virus
RNA viruses	Influenza C virus
RNA viruses	Influenza A(H1N1)pdm09 virus
RNA viruses	Influenza B virus (B/Victoria)
RNA viruses	Influenza B virus (B/Yamagata)
RNA viruses	Measles morbillivirus
RNA viruses	Mumps orthorubulavirus
RNA viruses	Rhinovirus
RNA viruses	Rhinovirus A
RNA viruses	Rhinovirus B
RNA viruses	Rhinovirus C
RNA viruses	Rubella virus
RNA viruses	SARS-CoV-2
Gram positive bacteria	*Corynebacterium diphtheriae*
Gram positive bacteria	*Mycobacterium asiaticum*
Gram positive bacteria	*Mycobacterium avium*
Gram positive bacteria	*Mycobacterium avium complex*
Gram positive bacteria	*Mycobacterium celatum*
Gram positive bacteria	*Mycobacterium gordonae*
Gram positive bacteria	*Mycobacterium intracellulare*
Gram positive bacteria	*Mycobacterium kansasii*
Gram positive bacteria	*Mycobacterium malmoense*
Gram positive bacteria	*Nontuberculous mycobacteria*
Gram positive bacteria	*Mycobacterium scrofulaceum*
Gram positive bacteria	*Mycobacterium shimoidei*
Gram positive bacteria	*Mycobacterium simiae*
Gram positive bacteria	*Mycobacterium szulgai*
Gram positive bacteria	*Mycobacterium tuberculosis complex*
Gram positive bacteria	*Mycobacterium xenopi*
Gram positive bacteria	*Mycobacteroides abscessus*
Gram positive bacteria	*Mycobacterium chelonae-abscessus complex*
Gram positive bacteria	*Mycobacteroides chelonae*
Gram positive bacteria	*Mycolicibacterium fortuitum*
Gram positive bacteria	*Mycolicibacterium smegmatis*
Gram positive bacteria	*Nocardia*
Gram positive bacteria	*Nocardia abscessus*
Gram positive bacteria	*Nocardia africana*
Gram positive bacteria	*Nocardia asteroides*
Gram positive bacteria	*Nocardia brasiliensis*
Gram positive bacteria	*Nocardia concava*
Gram positive bacteria	*Nocardia cyriacigeorgica*
Gram positive bacteria	*Nocardia farcinica*
Gram positive bacteria	*Nocardia nova*
Gram positive bacteria	*Nocardia otitidiscaviarum*
Gram positive bacteria	*Nocardia terpenica*
Gram positive bacteria	*Parvimonas micra*
Gram positive bacteria	*Rhodococcus hoagii*
Gram positive bacteria	*Staphylococcus aureus*
Gram positive bacteria	*Streptococcus agalactiae*
Gram positive bacteria	*Streptococcus anginosus group*
Gram positive bacteria	*Streptococcus intermedius*
Gram positive bacteria	*Streptococcus pneumoniae*
Gram positive bacteria	*Streptococcus pyogenes*
Gram positive bacteria	*Tropheryma whipplei*
Gram positive bacteria	*Trueperella pyogenes*
Gram negative bacteria	*Acinetobacter baumannii*
Gram negative bacteria	*Acinetobacter junii*
Gram negative bacteria	*Acinetobacter ursingii*
Gram negative bacteria	*Bacteroides fragilis*
Gram negative bacteria	*Bordetella holmesii*
Gram negative bacteria	*Bordetella parapertussis*
Gram negative bacteria	*Bordetella pertussis*
Gram negative bacteria	*Brucella*
Gram negative bacteria	*Burkholderia cenocepacia*
Gram negative bacteria	*Burkholderia cepacia*
Gram negative bacteria	*Burkholderia cepacia complex*
Gram negative bacteria	*Burkholderia mallei*
Gram negative bacteria	*Burkholderia pseudomallei*
Gram negative bacteria	*Burkholderia contaminans*
Gram negative bacteria	*Burkholderia multivorans*
Gram negative bacteria	*Elizabethkingia anophelis*
Gram negative bacteria	*Elizabethkingia meningoseptica*
Gram negative bacteria	*Enterobacter cloacae complex*
Gram negative bacteria	*Escherichia coli*
Gram negative bacteria	*Fusobacterium necrophorum*
Gram negative bacteria	*Fusobacterium nucleatum*
Gram negative bacteria	*Haemophilus influenzae*
Gram negative bacteria	*Klebsiella aerogenes*
Gram negative bacteria	*Klebsiella oxytoca*
Gram negative bacteria	*Klebsiella pneumoniae*
Gram negative bacteria	*Klebsiella variicola*
Gram negative bacteria	*Legionella*
Gram negative bacteria	*Legionella bozemanae*
Gram negative bacteria	*Legionella pneumophila*
Gram negative bacteria	*Legionella longbeachae*
Gram negative bacteria	*Legionella micdadei*
Gram negative bacteria	*Moraxella catarrhalis*
Gram negative bacteria	*Neisseria meningitidis*
Gram negative bacteria	*Pasteurella multocida*
Gram negative bacteria	*Proteus mirabilis*
Gram negative bacteria	*Pseudomonas aeruginosa*
Gram negative bacteria	*Serratia marcescens*
Gram negative bacteria	*Stenotrophomonas maltophilia*
Mycoplasma, Chlamydia, etc	*Chlamydia pneumoniae*
Mycoplasma, Chlamydia, etc	*Chlamydia psittaci*
Mycoplasma, Chlamydia, etc	*Chlamydia trachomatis*
Mycoplasma, Chlamydia, etc	*Coxiella burnetii*
Mycoplasma, Chlamydia, etc	*Mycoplasma pneumoniae*
Mycoplasma, Chlamydia, etc	*Ureaplasma urealyticum*
Mycoplasma, Chlamydia, etc	*Ureaplasma parvum*
Fungi	*[Candida] glabrata*
Fungi	*Aspergillus flavus complex*
Fungi	*Aspergillus fumigatus*
Fungi	*Aspergillus niger complex*
Fungi	*Aspergillus terreus complex*
Fungi	*Candida albicans*
Fungi	*Candida orthopsilosis*
Fungi	*Candida parapsilosis*
Fungi	*Candida tropicalis*
Fungi	*Cryptococcus gattii*
Fungi	*Cryptococcus neoformans*
Fungi	*Fusarium*
Fungi	*Histoplasma capsulatum*
Fungi	*Lichtheimia*
Fungi	*Lichtheimia corymbifera*
Fungi	*Lichtheimia ramosa*
Fungi	*Meyerozyma guilliermondii*
Fungi	*Mucor irregularis*
Fungi	*Mucor racemosus*
Fungi	*Pichia kudriavzevii*
Fungi	*Pneumocystis jirovecii*
Fungi	*Rhizomucor*
Fungi	*Rhizomucor pusillus*
Fungi	*Rhizopus*
Fungi	*Rhizopus delemar*
Fungi	*Rhizopus microsporus*
Fungi	*Rhizopus oryzae*
Fungi	*Scedosporium*
Fungi	*Scedosporium apiospermum*
Fungi	*Scedosporium boydii*
Fungi	*Talaromyces marneffei*
Fungi	*Trichosporon asahii*

### Appendix 3. List of conventional microbiologic test available

**Table:**

Pathogen types	Conventional microbiologic test
Bacteria	Gram stain
Bacteria	Bacterial culture
Bacteria	Modified acid-fast bacilli stain for Nocardia
Bacteria	Urinary antigen for *Streptococcus pneumoniae* and *Legionella pneumophila*
Bacteria	PCR assays (*M. pneumoniae, C. pneumoniae, Ureaplasma urealyticum, and legionella pneumophila*)
Mycobacteria	Acid-fast bacilli smear stain
Mycobacteria	Mycobacterial culture
Mycobacteria	PCR assays for mycobacterium tuberculosis complex (GeneXpert)
Virus	Antigen test (influenza A, influenza B, SARS-CoV-2)
Virus	PCR assays (influenza A H1N1, influenza A H7N9, influenza A H3N2, influenza B, respiratory syncytial virus, parainfluenza virus, adenovirus, human rhinovirus, human coronavirus, human bocavirus, Epstein-Barr virus, herpes simplex virus, human cytomegalovirus, and SARS-CoV-2)
Fungi	Gram stain
Fungi	Fungal culture
Fungi	Gomori methenamine silver (GMS) staining for *P. jirovecii*
Fungi	PCR assays (*P. jirovecii*)
Fungi	Cryptococcus antigen tests
