# Supplementary material for: Myxovirus resistance protein A to differentiate between viral and non-viral respiratory infections in adults: a prospective study
Source: Emerg Microbes Infect. 2026 Jan 16;15(1):2614734. doi: 10.1080/22221751.2026.2614734 (PMC12818327; doi:10.1080/22221751.2026.2614734)
Supplement: Supplemental Material [file TEMI_A_2614734_SM1433.docx]

**SUPPLEMENTAL MATERIALS**

**Manuscript Title:**

Myxovirus resistance protein A to differentiate between viral and non-viral respiratory infections in adults: a prospective study

**Authors:**

Mengwei Yan^1*^, Nengyong Wang^2*^, Liping Yang^2^, Xiaoqi Zhang^3^, Yong Zhang^3^, Weixia Xuan^4^, Xiaoju Zhang^4^, Gang Liu^5^, Herong Wang^6^, Yao Qing^7^, Yeming Wang^1†^, Bin Cao^1†^

**Affiliations:**

^1^ Capital Medical University, National Center for Respiratory Medicine, State Key Laboratory of Respiratory Health and Multimorbidity, National Clinical Research Center for Respiratory Diseases, Institute of Respiratory Medicine, Chinese Academy of Medical Sciences, New Cornerstone Science Laboratory, Department of Pulmonary and Critical Care Medicine, Center of Respiratory Medicine, China-Japan Friendship Hospital, Beijing 100084, China;

^2^ Department of Clinical Laboratory, Guangyuan Central Hospital, Sichuan Province, China;

^3^ Department of Respiratory and Critical Care Medicine, Weifang Second People's Hospital, Shandong Province, China;

^4^ Department of Respiratory and Critical Care Medicine; Henan Provincial People’s Hospital, Zhengzhou, Henan, China;

^5^ Department of Clinical Laboratory, Haihe Hospital, Tianjin University, Tianjin, China;

^6^ Department of Respiratory and Critical Care, Haihe Hospital, Tianjin University, Tianjin, China;

^7^ Zybio Inc., Chongqing, China

**Co-corresponding Authors:**

Dr. Yeming Wang. Address: No 2, East Yinghua Road, Chaoyang District, Beijing, China. 100020. E-mail: wwyymm_love@163.com (Y. Wang). Tel: 8610-84206269.

Prof. Bin Cao. Address: No 2, East Yinghua Road, Chaoyang District, Beijing, China. 100020. E-mail: caobin_ben@163.com (B. Cao). Tel: 8610-84206264.

**Supplementary Table 1.** Baseline Characteristics by pathogen group.

| **Characteristic** | **Total**  **(n=518)** | **Viral-detected group (n=325)** | **Bacterial/Fungal-detected group (n=131)** | **No pathogen detected group (n=62)** | ***Typical Bacteria-detected group ^a^***  ***(n=75)*** | ***Atypical Bacteria-detected group ^a^***  ***(n=22)*** | ***Mycobacterial/Fungal-detected group ^a^***  ***(n=13)*** | ***Mixed Pathogens-detected group ^a, b^ (n=21)*** | **P value ^c^** |
| --- | --- | --- | --- | --- | --- | --- | --- | --- | --- |
| *Median Age, years* | 44 (31-67) | 40 (29-62) | 62 (40-70) | 39 (29-66) | 59 (37-68) | 47 (36-61) | 70 (60-76) | 68 (63-73) | **<0.001** |
| *Female sex* | 268 (51.7) | 168 (51.7) | 61 (46.6) | 39 (62.9) | 35 (46.7) | 12 (54.5) | 6 (46.2) | 8 (38.1) | 0.32 |
| *Median duration of symptoms, days* | 3 (1-8) | 3 (1-7) | 6 (3-11) | 4 (1-7) | 5 (2-10) | 9 (5-11) | 7 (5-14) | 11 (5-19) | **<0.001** |
| *Comorbidities* | 229 (44.2) | 116 (35.7) | 86 (65.6) | 27 (43.5) | 49 (65.3) | 8 (36.4) | 10 (76.9) | 19 (90.5) | **<0.001** |
| Chronic lung diseases | 85 (16.4) | 34 (10.5) | 42 (32.1) | 9 (14.5) | 22 (29.3) | 4 (18.2) | 3 (23.1) | 13 (61.9) | **<0.001** |
| Diabetes mellitus | 54 (10.4) | 31 (9.5) | 16 (12.2) | 7 (11.3) | 8 (10.7) | 2 (9.1) | 3 (23.1) | 3 (14.3) | 0.40 |
| Cancer | 39 (7.5) | 25 (7.7) | 10 (7.6) | 4 (6.5) | 5 (6.7) | 2 (9.1) | 1 (7.7) | 2 (9.5) | 0.98 |
| Chronic heart Failure | 9 (1.7) | 7 (2.2) | 2 (1.5) | 0 | 0 | 0 | 1 (7.7) | 1 (4.8) | 0.95 |
| Hypertension | 98 (18.9) | 51 (15.7) | 33 (25.2) | 14 (22.6) | 16 (21.3) | 4 (18.2) | 6 (46.2) | 7 (33.3) | **0.02** |
| Coronary atherosclerotic heart  disease | 36 (6.9) | 20 (6.2) | 15 (11.5) | 1 (1.6) | 8 (10.7) | 2 (9.1) | 3 (23.1) | 2 (9.5) | 0.06 |
| Chronic kidney Disease | 14 (2.7) | 9 (2.8) | 3 (2.3) | 2 (3.2) | 2 (2.7) | 0 | 0 | 1 (4.8) | >0.99 |
| Chronic liver Disease | 5 (1.0) | 3 (0.9) | 2 (1.5) | 0 | 2 (2.7) | 0 | 0 | 0 | 0.95 |
| Autoimmune rheumatic disease | 21 (4.1) | 11 (3.4) | 5 (3.8) | 5 (8.1) | 3 (4.0) | 0 | 0 | 2 (9.5) | 0.82 |
| *Immunosuppression* | 49 (9.5) | 33 (10.2) | 10 (7.6) | 6 (9.7) | 5 (6.7) | 1 (4.5) | 2 (15.4) | 2 (9.5) | 0.94 |
| Active malignancy | 27 (5.2) | 18 (5.5) | 5 (3.8) | 4 (6.5) | 3 (4.0) | 1 (4.5) | 1 (7.7) | 0 | … |
| Solid organ transplantation | 3 (0.6) | 2 (0.6) | 1 (0.8) | 0 | 1 (1.3) | 0 | 0 | 0 | … |
| Receiving corticosteroid therapy | 15 (2.9) | 10 (3.1) | 4 (3.1) | 1 (1.6) | 0 | 0 | 2 (15.4) | 2 (9.5) | … |
| Receiving immunosuppressive drugs | 18 (3.5) | 13 (4.0) | 4 (3.1) | 1 (1.6) | 3 (4.0) | 0 | 1 (7.7) | 0 | … |
| *Chronic infections* | 17 (3.3) | 12 (3.7) | 4 (3.1) | 1 (1.6) | 1 (1.3) | 0 | 2 (15.4) | 1 (4.8) | 0.90 |
| TB | 5 (1.0) | 3 (0.9) | 2 (1.5) | 0 | 0 | 0 | 1 (7.7) | 1 (4.8) | … |
| HBV | 10 (1.9) | 7 (2.2) | 2 (1.5) | 1 (1.6) | 1 (1.3) | 0 | 1 (7.7) | 0 | … |
| Other ^d^ | 2 (0.4) | 2 (0.6) | 0 | 0 | 0 | 0 | 0 | 0 | … |
| *Antiviral treatment before enrollment* | 65 (12.5) | 43 (13.2) | 15 (11.5) | 7 (11.3) | 10 (13.3) | 0 | 2 (15.4) | 3 (14.3) | 0.61 |
| *Antibiotic treatment before enrollment* | 185 (35.7) | 98 (30.2) | 66 (50.4) | 21 (33.9) | 33 (44.0) | 12 (54.5) | 10 (76.9) | 11 (52.4) | **<0.001** |
| *Glucocorticoid treatment before enrollment* | 79 (15.3) | 49 (15.1) | 23 (17.6) | 7 (11.3) | 11 (14.7) | 2 (9.1) | 3 (23.1) | 7 (33.3) | 0.51 |
| *Site of enrollment* |  |  |  |  |  |  |  |  | **<0.001** |
| Outpatients | 287 (55.4) | 218 (67.1) | 31 (23.7) | 38 (61.3) | 27 (36.0) | 4 (18.2) | 0 | 0 | … |
| General ward | 144 (27.8) | 55 (16.9) | 69 (52.7) | 20 (32.3) | 31 (41.3) | 15 (68.2) | 8 (61.5) | 15 (71.4) | … |
| ICU | 87 (16.8) | 52 (16.0) | 31 (23.7) | 4 (6.5) | 17 (22.7) | 3 (13.6) | 5 (38.5) | 6 (28.6) | … |
| *Requires ventilation or supplemental oxygen* | 192 (37.1) | 90 (27.7) | 88 (67.2) | 14 (22.6) | 40 (53.3) | 15 (68.2) | 12 (92.3) | 21 (100.0) | **<0.001** |
| Requiring supplemental oxygen | 92 (17.8) | 31 (9.5) | 51 (38.9) | 10 (16.1) | 18 (24.0) | 12 (54.5) | 8 (61.5) | 13 (61.9) | … |
| Non-invasive mechanical ventilation or HFNC | 65 (12.5) | 35 (10.8) | 28 (21.4) | 2 (3.2) | 16 (21.3) | 3 (13.6) | 3 (23.1) | 6 (28.6) | … |
| Mechanical (invasive) ventilation | 35 (6.8) | 24 (7.4) | 9 (6.9) | 2 (3.2) | 6 (8.0) | 0 | 1 (7.7) | 2 (9.5) | … |
| *Final diagnosis* |  |  |  |  |  |  |  |  | **<0.001** |
| URTI | 300 (57.9) | 226 (69.5) | 31 (23.7) | 43 (69.4) | 27 (36.0) | 4 (18.2) | 0 | 0 | … |
| LRTI | 218 (42.1) | 99 (30.5) | 100 (76.3) | 19 (30.6) | 48 (64.0) | 18 (81.8) | 13 (100.0) | 21 (100.0) | … |
| *Antibiotic treatment after enrollment* | 235 (45.4) | 113 (34.8) | 99 (75.6) | 23 (37.1) | 51 (68.0) | 19 (86.4) | 11 (84.6) | 18 (85.7) | **<0.001** |
| *Median time in hospital, days* ^e^ | 13 (9-20) | 15 (9-22) | 13 (8-18) | 11 (7-12) | 13 (8-19) | 9 (8-10) | 14 (10-23) | 17 (12-27) | 0.13 |
| *In-hospital mortality* ^e^ | 14/231 (6.1) | 11/107 (10.3) | 2/100 (2.0) | 1/24 (4.2) | 2/48 (4.2) | 0/18 | 0/13 | 0/21 | **0.008** |

Data are n (%) or median (IQR). TB=tuberculosis. HBV=hepatitis B virus. ICU=intensive care unit. HFNC=high-flow nasal cannula oxygen therapy. URTI=upper respiratory tract infection. LRTI=lower respiratory tract infection.

^a^ The Typical Bacteria-detected group, Atypical Bacteria-detected group, Mycobacterial/Fungal-detected group, and Mixed Pathogens-detected group are all subgroups within the Bacterial/Fungal-detected group.

^b^ Patients in whom respiratory viruses were not detected but more than one type of other causative pathogen was identified (e.g., bacterial–fungal co-detection) were classified as the mixed pathogens-detected group. This group included: 1 bacterial–mycobacterial co-detection, 16 bacterial–fungal co-detections, 2 bacterial–fungal–mycobacterial co-detections, 1 fungal–bacterial co-detection, and 1 fungal–mycobacterial co-detection.

^c^ p values are of differences between Viral-detected group and Bacteria/Fungal-detected group.

^d^ Among the other patients with chronic infections, one was infected with Treponema Pallidum and the other was infected with aspergillus.

^e^ Calculations were based on hospitalized patients.

**Supplementary Table 2.** Baseline Characteristics of patients with URTI and LRTI.

| **Characteristic** | **URTI (n=300)** | **LRTI (n=218)** |
| --- | --- | --- |
| *Median Age, years* | 34 (27-46) | 66 (53-73) |
| *Female sex* | 187 (62.3) | 81 (37.2) |
| *Median duration of symptoms, days* | 2 (1-3) | 8 (5-12) |
| *Comorbidities* | 55 (18.3) | 174 (79.8) |
| Chronic lung diseases | 9 (3.0) | 76 (34.9) |
| Diabetes mellitus | 8 (2.7) | 46 (21.1) |
| Cancer | 5 (1.7) | 34 (15.6) |
| Chronic heart Failure | 2 (0.7) | 7 (3.2) |
| Hypertension | 14 (4.7) | 84 (38.5) |
| Coronary atherosclerotic heart disease | 2 (0.7) | 34 (15.6) |
| Chronic kidney Disease | 3 (1.0) | 11 (5.0) |
| Chronic liver Disease | 1 (0.3) | 4 (1.8) |
| Autoimmune rheumatic disease | 5 (1.7) | 16 (7.3) |
| *Immunosuppression* | 7 (2.3) | 42 (19.3) |
| Active malignancy | 4 (1.3) | 23 (10.6) |
| Solid organ transplantation | 0 | 3 (1.4) |
| Receiving corticosteroid therapy | 1 (0.3) | 14 (6.4) |
| Receiving immunosuppressive drugs | 2 (0.7) | 16 (7.3) |
| *Chronic infections* | 2 (0.7) | 15 (6.9) |
| TB | 0 | 5 (2.3) |
| HBV | 2 (0.7) | 8 (3.7) |
| Other ^a^ | 0 | 2 (0.9) |
| *Antiviral treatment before enrollment* | 11 (3.7) | 54 (24.8) |
| *Antibiotic treatment before enrollment* | 22 (7.3) | 163 (74.8) |
| *Glucocorticoid treatment before enrollment* | 7 (2.3) | 72 (33.0) |
| *Site of enrollment* |  |  |
| Outpatients | 286 (95.3) | 1 (0.5) |
| General ward | 13 (4.3) | 131 (60.1) |
| ICU | 1 (0.3) | 86 (39.4) |
| *Requires ventilation or supplemental oxygen* | 6 (2.0) | 186 (85.3) |
| Requiring supplemental oxygen | 5 (1.7) | 87 (39.9) |
| Non-invasive mechanical ventilation or HFNC | 1 (0.3) | 64 (29.4) |
| Mechanical (invasive) ventilation | 0 | 35 (16.1) |
| *Median time in hospital, days* ^b^ | 15 (9-30) | 13 (8-20) |
| *In-hospital mortality* ^b^ | 0/14 | 14/217 (6.5) |
| *Antibiotic treatment after enrollment* | 62 (20.7) | 173 (79.4) |
| *Etiological diagnosis* |  |  |
| Viral-detected | 226 (75.3) | 99 (45.4) |
| Bacteria/Fungal-detected | 31 (10.3) | 100 (45.9) |
| Typical Bacteria-detected | 27 (9.0) | 48 (22.0) |
| Atypical Bacteria-detected | 4 (1.3) | 18 (8.3) |
| Mycobacterial/ Fungal-detected | 0 | 13 (6.0) |
| Mixed Pathogens-detected ^c^ | 0 | 21 (9.6) |
| No pathogen detected | 43 (14.3) | 19 (8.7) |

Data are n (%) or median (IQR). URTI=upper respiratory tract infection. LRTI=lower respiratory tract infection. TB=tuberculosis. HBV=hepatitis B virus. ICU=intensive care unit. HFNC=high-flow nasal cannula oxygen therapy.

^a^ Among the other patients with chronic infections, one was infected with Treponema Pallidum and the other was infected with aspergillus.

^b^ Calculations were based on hospitalized patients.

^c^ Patients in whom respiratory viruses were not detected but more than one type of other causative pathogen was identified (e.g., bacterial–fungal co-detection) were classified as the mixed pathogens-detected group. This group included: 1 bacterial–mycobacterial co-detection, 16 bacterial–fungal co-detections, 2 bacterial–fungal–mycobacterial co-detections, 1 fungal–bacterial co-detection, and 1 fungal–mycobacterial co-detection.

**Supplementary Table 3.** MxA levels by specific pathogens detected.

| **Pathogen** |  | **MxA (ng/ml)** | | | | |
| --- | --- | --- | --- | --- | --- | --- |
|  | **n** | **Median** | **Minimum** | **25^th^ percentile** | **75^th^ percentile** | **Maximum** |
| ***Viral-detected*** | **325** | **123.63** | **<5.00** | **56.43** | **189.57** | **>1000.00** |
| ***Single virus-detected*** | **298** | **125.85** | **<5.00** | **61.70** | **198.43** | **>1000.00** |
| Flu A | 65 | 118.15 | <5.00 | 82.76 | 176.44 | 491.67 |
| Flu B | 51 | 157.92 | <5.00 | 130.94 | 208.02 | 760.69 |
| SARS-CoV-2 | 106 | 127.29 | <5.00 | 71.71 | 238.30 | 420.70 |
| Adenovirus | 18 | 158.17 | 5.58 | 27.11 | 446.01 | >1000.00 |
| RSV | 26 | 48.40 | 7.32 | 16.36 | 119.95 | >1000.00 |
| Rhinovirus | 14 | 40.18 | <5.00 | 11.72 | 89.39 | 620.16 |
| Parainfluenza virus | 3 | 239.24 | 9.49 | 9.49 | n/a | 431.93 |
| Human coronaviruses | 6 | 39.33 | 18.11 | 18.64 | 244.81 | 573.92 |
| Human metapneumovirus | 8 | 57.55 | 6.15 | 36.47 | 103.23 | 229.13 |
| Enterovirus D68 | 1 | 16.44 | n/a | n/a | n/a | n/a |
| ***Multiple viruses-detected*** | **27** | **73.47** | **9.07** | **18.75** | **140.20** | **236.11** |
| RSV and Cytomegalovirus | 1 | 42.85 | n/a | n/a | n/a | n/a |
| Rhinovirus and Parainfluenza virus | 2 | 74.89 | 73.47 | 73.47 | n/a | 76.30 |
| Rhinovirus and Human metapneumovirus | 1 | 128.57 | n/a | n/a | n/a | n/a |
| Parainfluenza virus and Enterovirus | 1 | 14.29 | n/a | n/a | n/a | n/a |
| Parainfluenza virus and RSV | 1 | 15.85 | n/a | n/a | n/a | n/a |
| Flu A and RSV | 3 | 123.67 | 70.87 | 70.87 | n/a | 163.35 |
| Flu A and Rhinovirus | 1 | 130.85 | n/a | n/a | n/a | n/a |
| Flu A and Parainfluenza virus | 1 | 236.11 | n/a | n/a | n/a | n/a |
| Flu A and Cytomegalovirus | 1 | 16.26 | n/a | n/a | n/a | n/a |
| Flu A and SARS-CoV-2 | 2 | 154.90 | 140.20 | 140.20 | n/a | 169.60 |
| Flu A and Flu B | 2 | 123.33 | 62.55 | 62.55 | n/a | 184.10 |
| SARS-CoV-2 and RSV | 2 | 29.80 | 21.54 | 21.54 | n/a | 38.06 |
| SARS-CoV-2 and Rhinovirus | 1 | 147.09 | n/a | n/a | n/a | n/a |
| SARS-CoV-2 and Rhinovirus and  Cytomegalovirus | 1 | 9.07 | n/a | n/a | n/a | n/a |
| SARS-CoV-2 and Cytomegalovirus | 2 | 52.75 | 30.45 | 52.75 | n/a | 75.05 |
| SARS-CoV-2 and Human coronaviruses | 2 | 127.70 | 105.12 | 127.70 | n/a | 150.28 |
| Flu B and Parainfluenza virus | 1 | 18.75 | n/a | n/a | n/a | n/a |
| Flu B and SARS-CoV-2 | 1 | 13.96 | n/a | n/a | n/a | n/a |
| Flu B and SARS-CoV-2 and RSV | 1 | 16.9 | n/a | n/a | n/a | n/a |
| ***Viral-mono-detected ^a^*** | **205** | **136.28** | **<5.00** | **85.68** | **193.46** | **>1000.00** |
| ***Viral-mixed-detected ^b^*** | **120** | **68.97** | **<5.00** | **18.73** | **167.14** | **>1000.00** |
| ***Bacterial/Fungal-detected*** | **131** | **15.93** | **<5.00** | **9.89** | **38.06** | **278.06** |
| ***Bacteria-detected*** | **97** | **18.66** | **<5.00** | **10.56** | **40.86** | **278.06** |
| ***Typical Bacteria*** | 75 | 15.30 | <5.00 | 9.81 | 28.43 | 150.75 |
| Haemophilus influenzae | 23 | 15.70 | 6.62 | 14.95 | 42.06 | 150.75 |
| Acinetobacter baumannii | 6 | 10.29 | 6.06 | 7.11 | 18.59 | 23.68 |
| Pseudomonas aeruginosa | 6 | 27.14 | 9.02 | 13.68 | 36.25 | 65.29 |
| Streptococcus pneumoniae | 4 | 13.86 | 7.40 | 8.65 | 35.33 | 85.35 |
| Staphylococcus aureus | 3 | 19.97 | 13.80 | 16.89 | 25.86 | 31.74 |
| Moraxella catarrhalis | 2 | 10.17 | 8.23 | 10.17 | n/a | 12.11 |
| Klebsiella pneumoniae | 1 | 13.60 | n/a | n/a | n/a | n/a |
| Streptococcus pyogenes | 1 | 11.89 | n/a | n/a | n/a | n/a |
| Fusobacterium necrophorum | 1 | 23.14 | n/a | n/a | n/a | n/a |
| Nocardia abscessus | 1 | 30.67 | n/a | n/a | n/a | n/a |
| Haemophilus influenzae, Acinetobacter baumannii | 2 | 10.68 | 9.75 | 10.68 | n/a | 11.60 |
| Haemophilus influenzae, Escherichia coli | 1 | 9.67 | n/a | n/a | n/a | n/a |
| Haemophilus influenzae, Streptococcus pneumoniae | 1 | 10.36 | n/a | n/a | n/a | n/a |
| Streptococcus pyogenes, Streptococcus pneumoniae | 1 | 30.17 | n/a | n/a | n/a | n/a |
| Moraxella catarrhalis, Streptococcus pneumoniae | 1 | 9.16 | n/a | n/a | n/a | n/a |
| Moraxella catarrhalis, Fusobacterium necrophorum | 1 | 18.80 | n/a | n/a | n/a | n/a |
| Corynebacterium striatum, Pseudomonas aeruginosa | 1 | 28.43 | n/a | n/a | n/a | n/a |
| Corynebacterium striatum, Streptococcus pneumoniae | 1 | <5.00 | n/a | n/a | n/a | n/a |
| Pseudomonas aeruginosa, Acinetobacter baumannii | 1 | 21.43 | n/a | n/a | n/a | n/a |
| Pseudomonas aeruginosa, Staphylococcus aureus | 1 | 56.69 | n/a | n/a | n/a | n/a |
| Pseudomonas aeruginosa, Escherichia coli, Klebsiella pneumoniae, Staphylococcus aureus, Stenotrophomonas maltophilia, Acinetobacter baumannii | 1 | 46.05 | n/a | n/a | n/a | n/a |
| Pseudomonas aeruginosa, Stenotrophomonas maltophilia, Burkholderia cepacian, Streptococcus pneumoniae | 1 | 9.89 | n/a | n/a | n/a | n/a |
| Haemophilus influenzae, Streptococcus pneumoniae, Escherichia coli | 1 | 11.32 | n/a | n/a | n/a | n/a |
| Haemophilus influenzae, Staphylococcus aureus | 1 | 14.18 | n/a | n/a | n/a | n/a |
| Haemophilus influenzae, Pseudomonas aeruginosa | 1 | 9.34 | n/a | n/a | n/a | n/a |
| Stenotrophomonas maltophilia, Acinetobacter baumannii | 1 | <5.00 | n/a | n/a | n/a | n/a |
| Acinetobacter baumannii, Burkholderia cepacian, Corynebacterium striatum, Pseudomonas aeruginosa, Klebsiella pneumoniae | 1 | 24.94 | n/a | n/a | n/a | n/a |
| Escherichia coli, Serratia marcescens | 1 | 11.96 | n/a | n/a | n/a | n/a |
| Klebsiella pneumoniae, Elizabethkingia anophelis | 1 | 16.05 | n/a | n/a | n/a | n/a |
| Klebsiella pneumoniae, Acinetobacter baumannii | 1 | 19.25 | n/a | n/a | n/a | n/a |
| Klebsiella pneumoniae, Escherichia coli, Acinetobacter baumannii | 1 | 16.31 | n/a | n/a | n/a | n/a |
| Klebsiella pneumoniae, Pseudomonas aeruginosa, Escherichia coli | 1 | 29.92 | n/a | n/a | n/a | n/a |
| Klebsiella pneumoniae, Pseudomonas aeruginosa, Enterobacter cloacae complex, Acinetobacter baumannii | 1 | 6.19 | n/a | n/a | n/a | n/a |
| Staphylococcus aureus, Klebsiella pneumoniae | 1 | 8.05 | n/a | n/a | n/a | n/a |
| Staphylococcus aureus, Klebsiella pneumoniae, Klebsiella aerogenes, Acinetobacter baumannii | 1 | 9.09 | n/a | n/a | n/a | n/a |
| Burkholderia cepacian, Klebsiella pneumoniae, Acinetobacter baumannii | 1 | 19.75 | n/a | n/a | n/a | n/a |
| ***Atypical Bacteria*** | 22 | 60.88 | 9.76 | 23.44 | 114.84 | 278.06 |
| Mycoplasma pneumoniae | 16 | 83.36 | 10.74 | 23.44 | 157.37 | 278.06 |
| Chlamydia pneumoniae | 1 | 58.79 | n/a | n/a | n/a | n/a |
| Legionella pneumophila | 3 | 36.62 | 9.76 | 23.19 | 38.34 | 40.05 |
| Chlamydia psittaci | 2 | 65.21 | 42.99 | 65.21 | n/a | 87.42 |
| ***Mycobacterial-detected*** | **4** | **14.00** | **6.82** | **8.13** | **16.29** | **16.41** |
| Mycobacterium tuberculosis complex | 2 | 14.00 | 12.06 | 14.00 | n/a | 15.93 |
| Mycobacterium xenopi | 1 | 16.41 | n/a | n/a | n/a | n/a |
| Mycobacteroides chelonae | 1 | 6.82 | n/a | n/a | n/a | n/a |
| ***Fungal-detected*** | **9** | **31.33** | **7.90** | **14.69** | **88.86** | **276.32** |
| Pneumocystis jirovecii | 3 | 14.18 | 7.90 | 11.04 | 57.84 | 101.5 |
| Aspergillus fumigatus | 3 | 31.33 | 15.19 | 23.26 | 53.77 | 76.21 |
| Aspergillus flavus complex | 2 | 165.10 | 53.88 | 165.10 | n/a | 276.32 |
| Rhizopus microsporus | 1 | 20.65 | n/a | n/a | n/a | n/a |
| ***Mixed Pathogens-detected*** | **21** | **11.56** | **<5.00** | **7.70** | **16.00** | **121.22** |
| ***Bacterial and Mycobacterial***  (Chlamydia psittaci and Mycobacteroides chelonae) | 1 | 5.94 | n/a | n/a | n/a | n/a |
| ***Bacterial and Fungal*** | 17 | 11.56 | <5.00 | 8.07 | 12.86 | 121.22 |
| Acinetobacter baumanni, Aspergillus flavus complex, Pneumocystis jirovecii | 1 | 9.31 | n/a | n/a | n/a | n/a |
| Acinetobacter baumannii, Aspergillus fumigatus | 1 | 8.07 | n/a | n/a | n/a | n/a |
| Nocardia farcinica, Aspergillus fumigatus, Aspergillus flavus complex | 1 | 7.06 | n/a | n/a | n/a | n/a |
| Klebsiella pneumoniae, Elizabethkingia anopheles, Aspergillus fumigatus | 1 | 7.33 | n/a | n/a | n/a | n/a |
| Klebsiella pneumoniae, Streptococcus pneumoniae, Pseudomonas aeruginosa, Aspergillus flavus complex | 1 | 6.62 | n/a | n/a | n/a | n/a |
| Klebsiella pneumoniae, Pseudomonas aeruginosa, Pneumocystis jirovecii | 1 | 81.53 | n/a | n/a | n/a | n/a |
| Klebsiella pneumoniae, Pneumocystis jirovecii | 1 | 13.88 | n/a | n/a | n/a | n/a |
| Klebsiella pneumoniae, Enterobacter cloacae complex, Aspergillus niger complex | 1 | <5.00 | n/a | n/a | n/a | n/a |
| Haemophilus influenzae, Moraxella catarrhalis, Klebsiella pneumoniae, Enterobacter cloacae complex, Acinetobacter baumannii, Aspergillus niger complex, Pneumocystis jirovecii | 1 | 12.44 | n/a | n/a | n/a | n/a |
| Haemophilus influenzae, Pseudomonas aeruginosa, Pneumocystis jirovecii, Aspergillus fumigatus | 1 | 18.11 | n/a | n/a | n/a | n/a |
| Stenotrophomonas maltophilia, Klebsiella pneumoniae, Aspergillus fumigatus | 1 | 11.82 | n/a | n/a | n/a | n/a |
| Pseudomonas aeruginosa, Serratia marcescens, Pneumocystis jirovecii | 1 | 12.29 | n/a | n/a | n/a | n/a |
| Pseudomonas aeruginosa, Aspergillus fumigatus | 1 | 11.56 | n/a | n/a | n/a | n/a |
| Pseudomonas aeruginosa, Aspergillus fumigatus, Pneumocystis jirovecii | 1 | 12.86 | n/a | n/a | n/a | n/a |
| Pseudomonas aeruginosa, Pneumocystis jirovecii | 2 | 9.21 | 8.55 | 9.21 | n/a | 9.87 |
| Pneumocystis jirovecii, Mycoplasma pneumoniae | 1 | 121.22 | n/a | n/a | n/a | n/a |
| ***Bacterial, Fungal, and Mycobacterial*** | 2 | 37.58 | 25.11 | 25.11 | n/a | 50.04 |
| Klebsiella pneumoniae, Pneumocystis jirovecii, Mycobacterium tuberculosis complex | 1 | 50.04 | n/a | n/a | n/a | n/a |
| Enterobacter cloacae complex, Stenotrophomonas maltophilia, Aspergillus fumigatus, and Mycobacterium kansasii | 1 | 25.11 | n/a | n/a | n/a | n/a |
| ***Fungal and Mycobacterial***  (Aspergillus fumigatus and Mycobacterium intracellulare) | 1 | 9.72 | n/a | n/a | n/a | n/a |
| ***No pathogen detected group*** | **62** | **57.74** | **<5.00** | **15.74** | **120.60** | **196.56** |
| ***Asymptomatic control group*** | **158** | **8.16** | **<5.00** | **<5.00** | **16.43** | **247.96** |

MxA=Myxovirus resistance protein A. Flu A=influenza A. Flu B=influenza B. RSV= respiratory syncytial virus.

^a^ Patients with only viral pathogens detected were classified as the viral-mono-detected group.

^b^ Patients with viral pathogens co-detected with other pathogens were classified as the viral-mixed-detected group.

**Supplemental Table 4.** Clinical characteristics of patients with RSV and Rhinovirus detected.

| **Characteristic** | **RSV-detected**  **(n=26)** | **Rhinovirus-detected**  **(n=14)** |
| --- | --- | --- |
| *Median Age, years* | 58 (27-70) | 49 (30-61) |
| *Female sex* | 12 (46.2) | 4 (28.6) |
| *Median duration of symptoms, days* | 3 (1-7) | 7 (5-9) |
| *Comorbidities* | 15 (57.7) | 7 (50.0) |
| Chronic lung diseases | 3 (11.5) | 4 (28.6) |
| Diabetes mellitus | 4 (15.4) | 1 (7.1) |
| Cancer | 9 (34.6) | 1 (7.1) |
| Chronic heart Failure | 1 (3.8) | 0 |
| Hypertension | 4 (15.4) | 3 (21.4) |
| Coronary atherosclerotic heart disease | 1 (3.8) | 2 (14.3) |
| Autoimmune rheumatic disease | 0 | 2 (14.3) |
| *Immunosuppression* | 10 (38.5) | 3 (21.4) |
| Active malignancy | 8 (30.8) | 1 (7.1) |
| Receiving corticosteroid therapy | 1 (3.8) | 1 (7.1) |
| Receiving immunosuppressive drugs | 2 (7.7) | 2 (14.3) |
| *Chronic infections* | 0 | 1 (7.1) |
| TB | 0 | 1 (7.1) |
| *Antiviral treatment before enrollment* | 8 (30.8) | 1 (7.1) |
| *Antibiotic treatment before enrollment* | 16 (61.5) | 8 (57.1) |
| *Glucocorticoid treatment before enrollment* | 6 (23.1) | 4 (28.6) |
| *Site of enrollment* |  |  |
| Outpatients | 11 (42.3) | 5 (35.7) |
| General ward | 13 (50.0) | 7 (50.0) |
| ICU | 2 (7.7) | 2 (14.3) |
| *Requires ventilation or supplemental oxygen* | 10 (38.5) | 7 (50.0) |
| Requiring supplemental oxygen | 6 (23.1) | 3 (21.4) |
| Non-invasive mechanical ventilation or HFNC | 4 (15.4) | 3 (21.4) |
| Mechanical (invasive) ventilation | 0 | 1 (7.1) |
| *Final diagnosis* |  |  |
| URTI | 15 (57.7) | 7 (50.0) |
| LRTI | 11 (42.3) | 7 (50.0) |
| *Median time in hospital, days* ^a^ | 20 (13-35) | 8 (7-15) |
| *In-hospital mortality* ^a^ | 0/15 | 2/9 (22.2) |
| *Antibiotic treatment after enrollment* | 12 (46.2) | 8 (57.1) |
| *Etiological diagnosis* |  |  |
| Viral mono-detected | 17 (65.4) | 5 (35.7) |
| Viral mixed detected | 9 (34.6) | 9 (64.3) |
| Viral-bacterial co-detections | 6 (23.1) | 7 (50.0) |
| Viral-bacterial-fungal co-detections | 1 (3.8) | 1 (7.1) |
| Viral-fungal co-detections | 2 (7.7) | 0 |
| Viral-bacterial-mycobacterial co-detection | 0 | 1 (7.1) |
| *Fever within 48 hours of blood sampling* | 18 (69.2) | 9 (64.3) |

Data are n (%) or median (IQR). RSV=respiratory syncytial viral. TB=tuberculosis. HBV=hepatitis B virus. ICU=intensive care unit. HFNC=high-flow nasal cannula oxygen therapy. URTI=upper respiratory tract infection. LRTI=lower respiratory tract infection.

^a^ Calculations were based on hospitalized patients.

**Supplemental Table 5.** Sensitivity analyses of the diagnostic performance of MxA for identifying viral infections in ARI patients using clinically informed etiological classification

|  | **Optimal Cutoff (ng/ml)** | **AUC**  **(95% CI)** | **Sensitivity**  **% (95% CI)** | **Specificity**  **% (95% CI)** | **PPV**  **% (95% CI)** | **NPV**  **% (95% CI)** |
| --- | --- | --- | --- | --- | --- | --- |
| Viral vs bacterial/fungal | 51 | 0.83  (0.78, 0.87) | 77.3  (72.7, 81.8) | 81.1  (73.7, 88.6) | 92.5  (89.4, 95.7) | 54.1  (46.3, 61.8) |
| Viral vs bacterial | 49 | 0.82  (0.77, 0.87) | 77.6  (73.0, 82.1) | 78.8  (70.1, 87.5) | 93.3  (90.3, 96.3) | 48.2  (39.9, 56.5) |
| Viral vs typical bacterial | 49 | 0.88  (0.84, 0.92) | 77.6  (73.0, 82.1) | 92.2  (85.6, 98.8) | 98.0  (96.3, 99.7) | 45.0  (36.5, 53.6) |

Data represent optimal cutoffs and corresponding diagnostic accuracy measures (95% CI) derived from receiver operating characteristic analyses in 3 comparisons:

(1) viral group vs. bacterial/fungal group;

(2) viral group vs. bacterial group (including both typical and atypical bacteria);

(3) viral group vs. typical bacterial group.

ARI=acute respiratory tract infection. MxA=Myxovirus resistance protein A. CI=confidence interval. AUC=area under the curve.

**Supplemental Table 6.** Multivariable logistic regression analysis evaluating MxA as an independent predictor of infection type (viral vs. bacterial/fungal)

| **Variable** | **aOR (95% CI)** | **P value** |
| --- | --- | --- |
| MxA | 1.02 (1.01, 1.03) | <0.001 |
| Age | 1.02 (1.00, 1.04) | 0.074 |
| Male | 1.52 (0.88, 2.66) | 0.136 |
| Duration of symptoms | 1.00 (0.97, 1.04) | 0.783 |
| Comorbidities | 0.81 (0.39, 1.74) | 0.584 |
| Immunosuppressed | 6.50 (2.79, 16.22) | <0.001 |
| Infection site of URTI | 6.73 (3.08, 15.22) | <0.001 |
| Fever within 48 hours of blood sampling | 1.11 (0.62, 1.98) | 0.735 |

aOR= adjusted odds ratio. MxA=myxovirus resistance protein A. URTI=upper respiratory tract infections.

**Supplemental Table 7.** Post hoc analyses of MxA for distinguishing viral infections from atypical bacterial infections and from other non-atypical infections.

| **Post hoc analyses** | **Number of patients classified as viral (%)** | **Optimal Cutoff (ng/ml)** | **AUC**  **(95% CI)** | **Sensitivity**  **% (95% CI)** | **Specificity**  **% (95% CI)** | **PPV**  **% (95% CI)** | **NPV**  **% (95% CI)** |
| --- | --- | --- | --- | --- | --- | --- | --- |
| Viral vs non-atypical infections ^a^ | 325/496 (65.5%) | 50 | 0.80  (0.76, 0.84) | 77.8  (73.3, 82.4) | 72.5  (65.8, 79.2) | 84.3  (80.2, 88.4) | 63.3  (56.5, 70.0) |
| Viral vs atypical bacterial infections | 325/347 (93.7%) | 105 | 0.65  (0.53, 0.77) | 58.5  (53.1, 63.8) | 77.3  (59.8, 94.8) | 97.4  (95.2, 99.7) | 11.2  (6.2, 16.2) |

Data represent optimal cutoffs and accuracy measures (95% CI) derived from receiver operating characteristic analyses in post hoc analyses of MxA for distinguishing viral infections from atypical bacterial and other non-atypical infections. MxA=Myxovirus resistance protein A. CI=confidence interval. AUC=area under the curve. NPV=negative predictive value. PPV=positive predictive value.

^a^ Non-atypical infections were defined as the non-viral-detected group excluding atypical bacteria.

**Supplemental Figure 1.** Technical roadmap on the accuracy of MxA in differentiating respiratory viral infections.


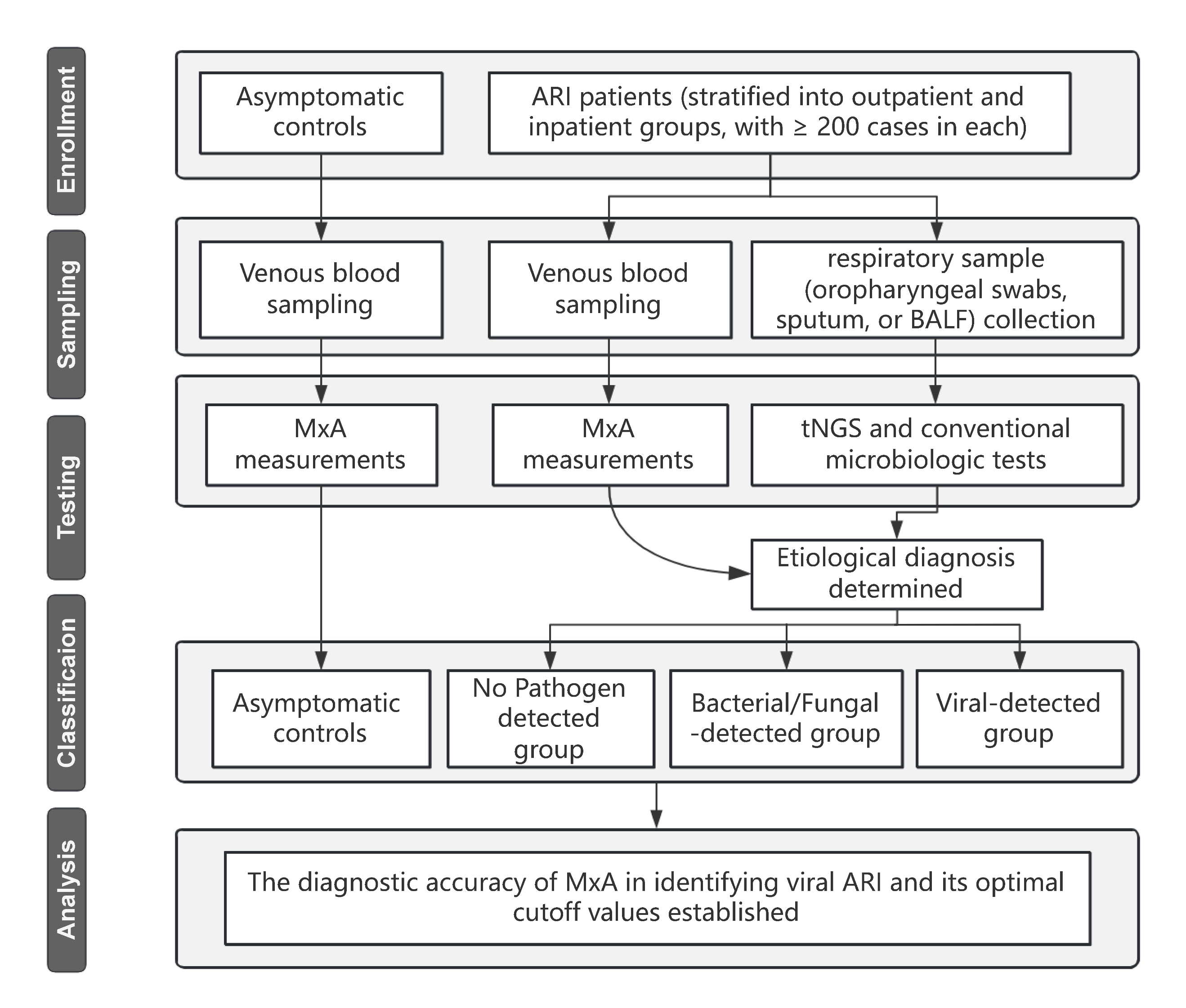


ARI=acute respiratory infections. BALF=bronchoalveolar lavage fluid. MxA=Myxovirus resistance protein A. tNGS= targeted next-generation sequencing.

**Supplemental Figure 2.** Etiological classification of acute upper respiratory tract infections.


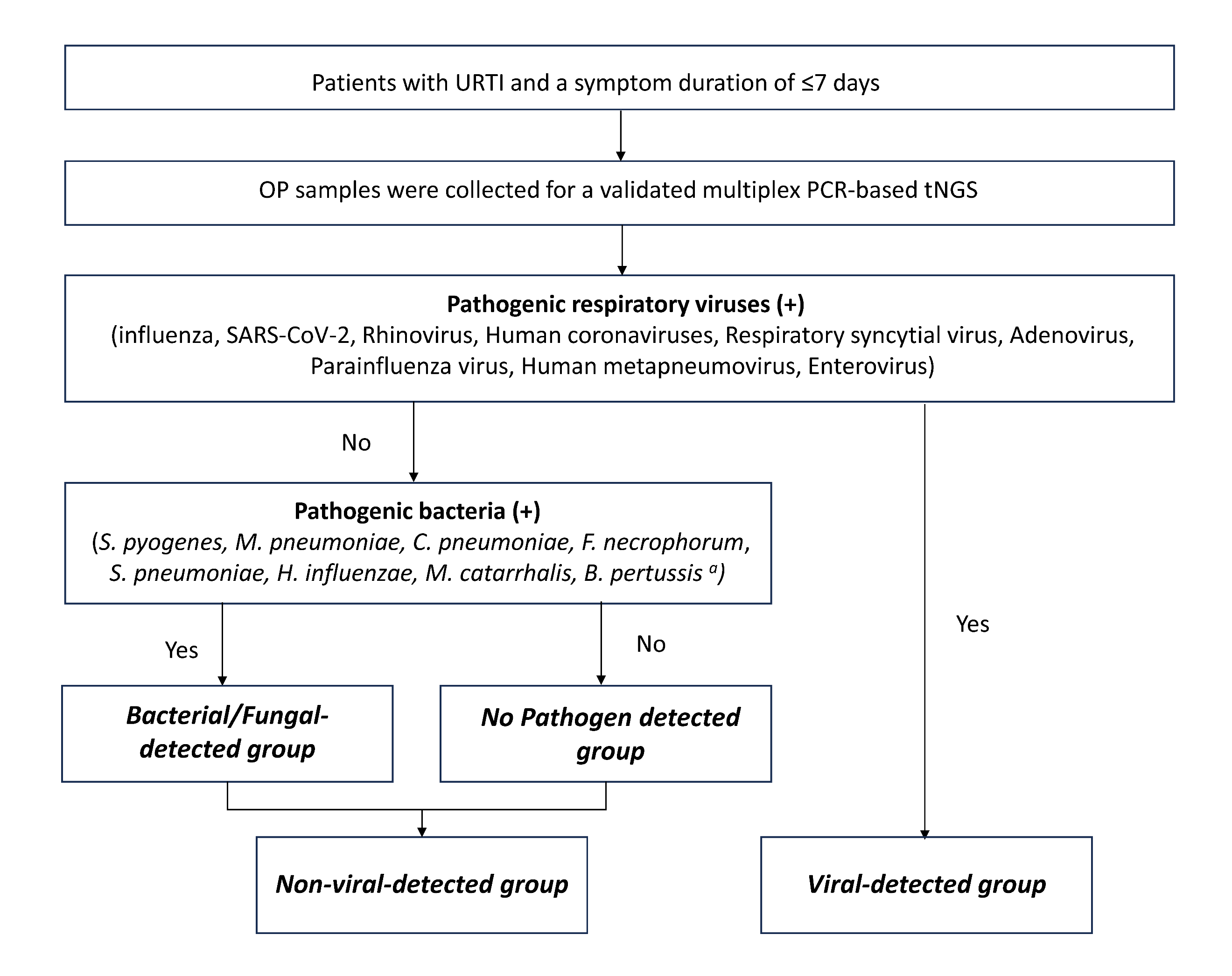


^a^ The pathogenic bacteria associated with URTI in this study were based on the reference index established in: Ann Intern Med. 2016 Mar 15;164(6):425-34. doi: 10.7326/M15-1840.

URTI=upper respiratory tract infection. OP=oropharyngeal swabs. tNGS=targeted next-generation sequencing. PCR=polymerase chain reaction.

**Supplemental Figure 3.** Etiological classification of lower respiratory tract infections.


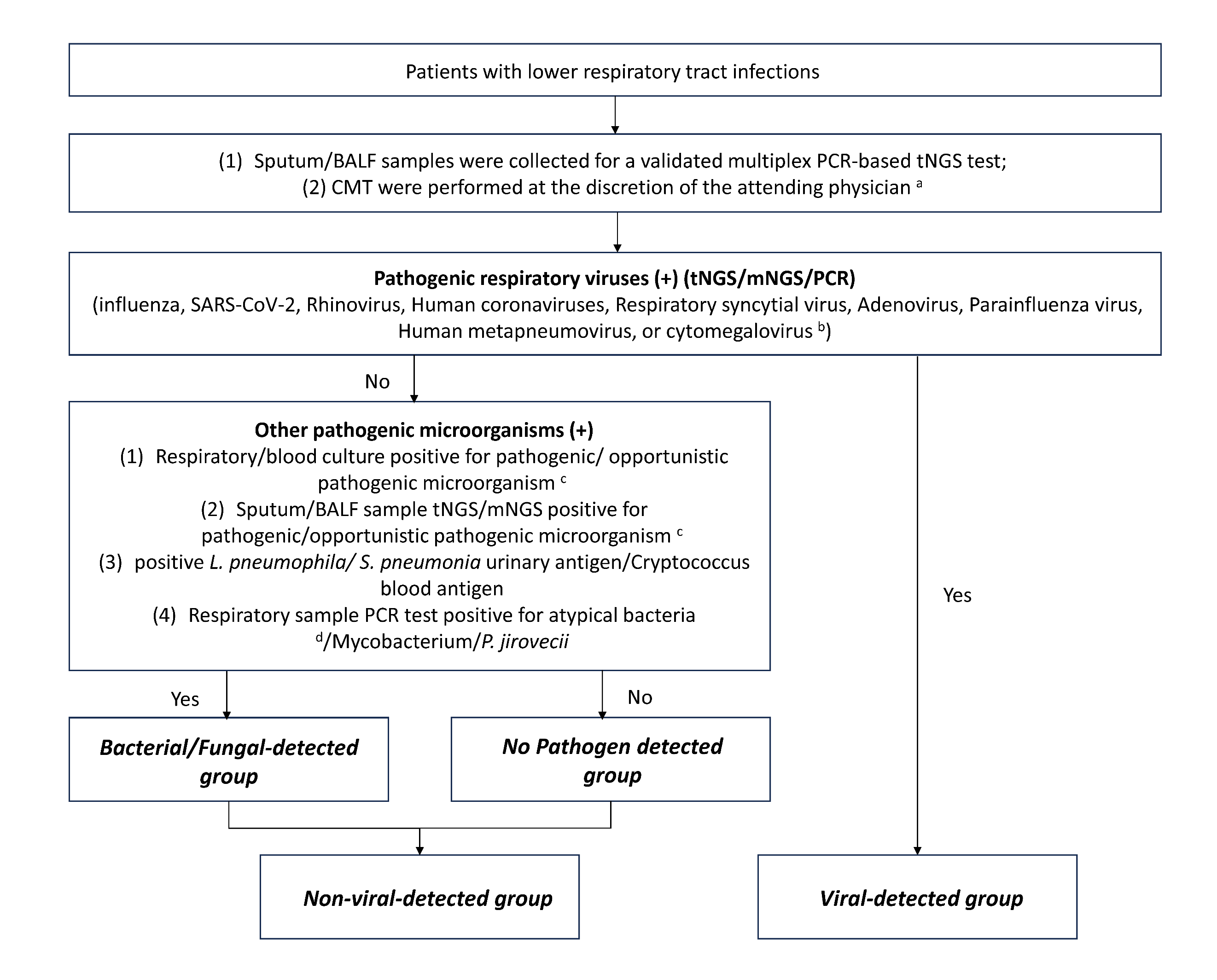


^a^ The available conventional microbiologic tests are shown in Appendix 2.

^b^ When both tNGS/mNGS and PCR were performed on sputum/BALF for respiratory virus detection and produced discrepant results, PCR findings were considered definitive. Cytomegalovirus detection should be considered pathogenic only in immunocompromised patients.

^c^ The definitions of pathogenic and opportunistic pathogenic microorganisms were based on the reference index established in: Zhonghua Jie He He Hu Xi Za Zhi. 2023 Apr 12;46(4):322-335. doi: 10.3760/cma.j.cn112147-20220701-00553

^d^ *M. pneumoniae, C. pneumoniae, U. urealyticum, and L. pneumophila.*

BALF=bronchoalveolar lavage fluid. CMT=Conventional microbiologic tests. tNGS=targeted next-generation sequencing. PCR=polymerase chain reaction. mNGS=metagenomic next generation sequencing.

**Supplemental Figure 4.** Box plot of MxA levels in patients with viral-mono-detected group and Viral-mixed-detected group.


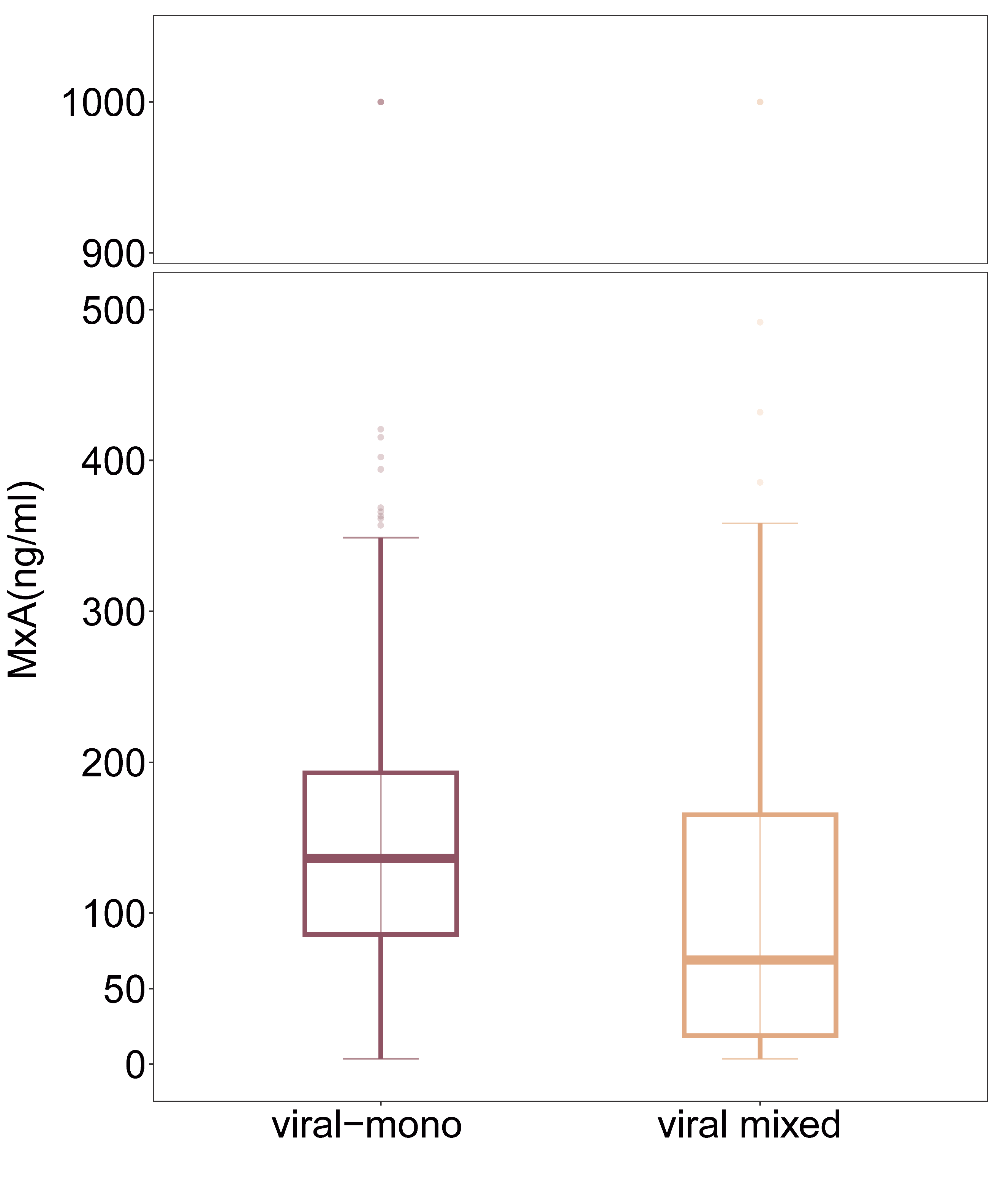


Patients with only viral pathogens detected were classified as the viral-mono-detected group, while patients with viral pathogens co-detected with other pathogens were classified as the viral-mixed-detected group. The p-value for the comparison between the viral-mono-detected and viral-mixed-detected group is <0.001.

Boxes indicate median and interquartile range, with whiskers indicate 1.5 times of IQR, and dots marking outliers beyond the whiskers.

MxA=Myxovirus resistance protein A.

**Supplemental Figure 5.** ROC curve of MxA to discriminate between viral and asymptomatic controls.

**
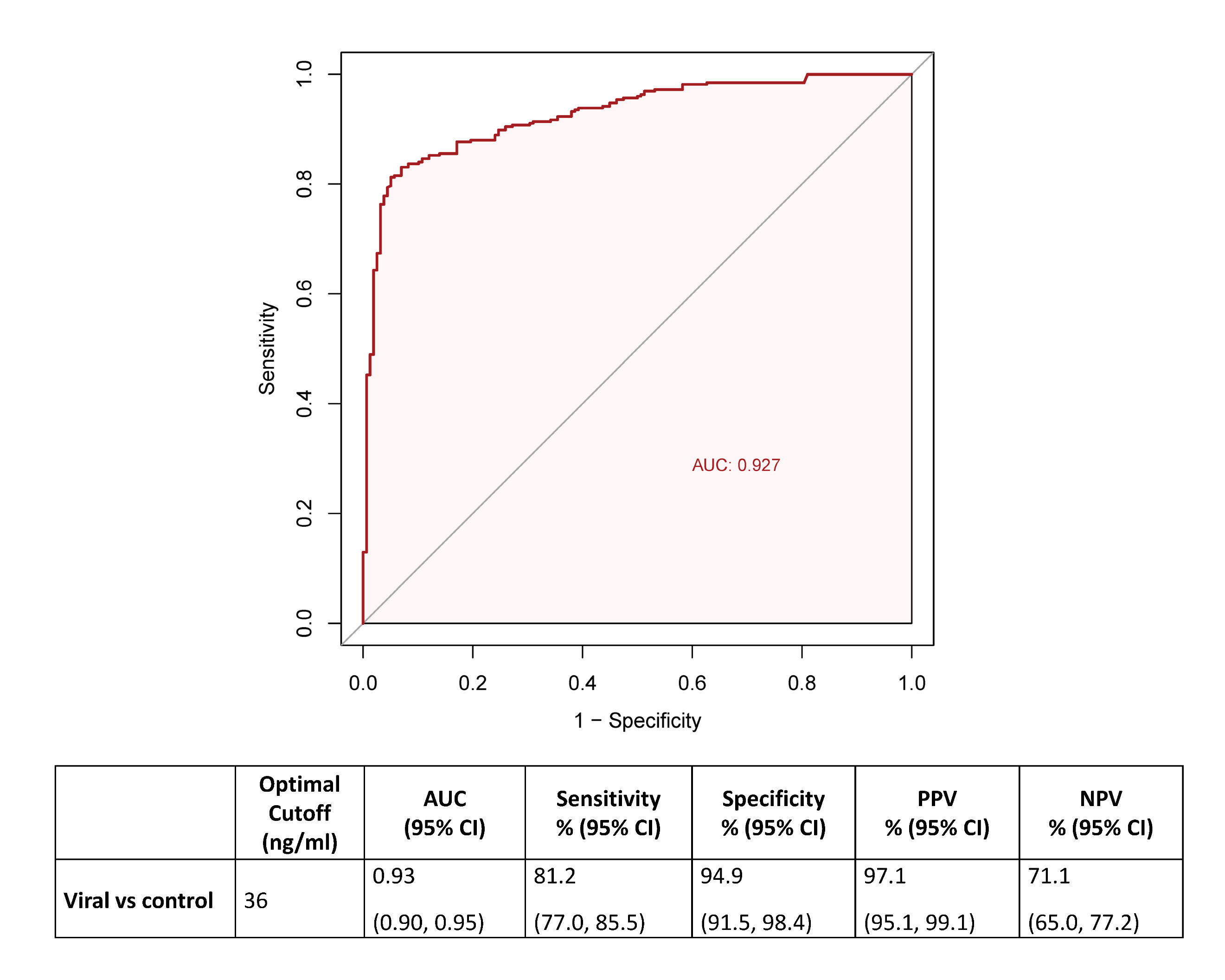
**

This figure presents the ROC curve of MxA for distinguishing viral infections from asymptomatic controls, with the corresponding AUC and optimal cutoff indicated.

ROC=receiver operating characteristic. MxA=Myxovirus resistance protein A. AUC=area under the curve. CI=confidence interval.

**Supplemental Figure 6.** Box plots comparing MxA levels between febrile and afebrile patients with respiratory viral infections within 48 hours of MxA testing.

**
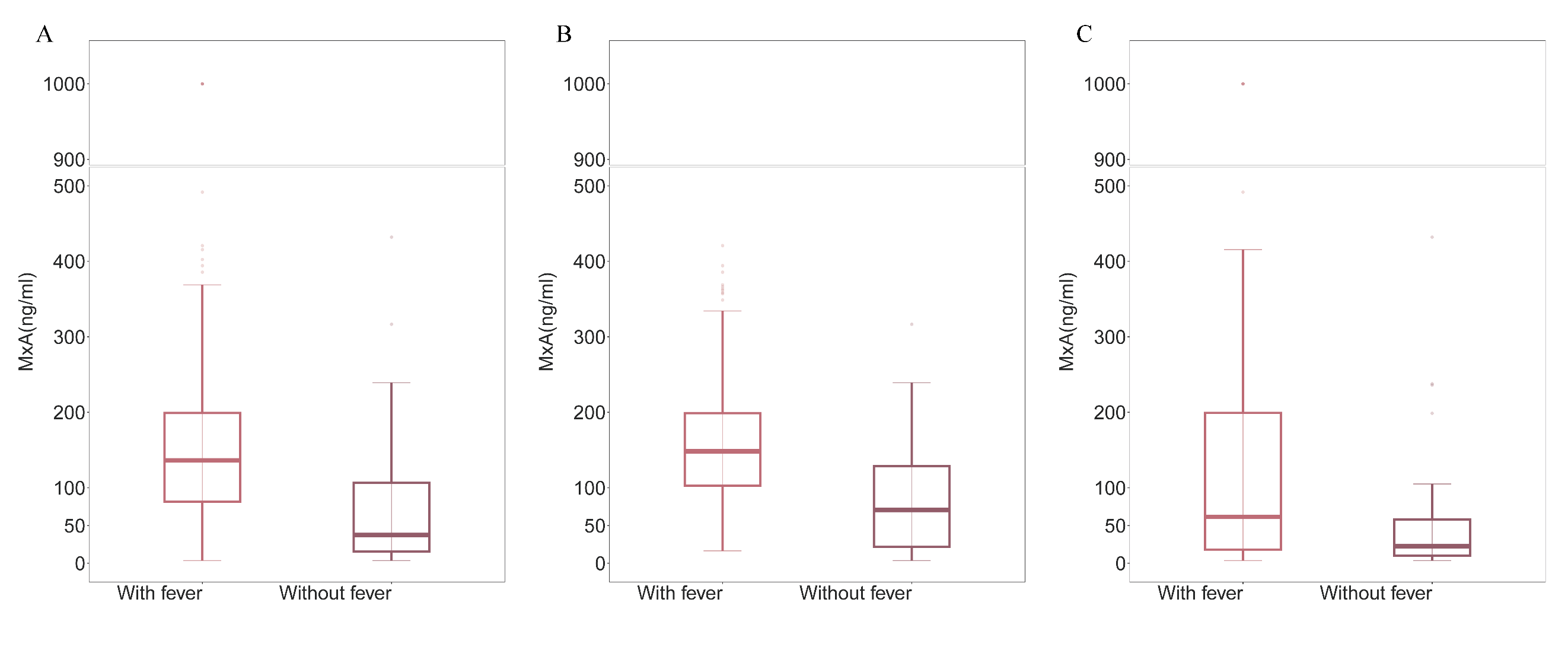
**

(A) All viral infections: median 136.0 ng/mL (IQR, 81.5–199.0) vs 37.4 ng/mL (IQR, 15.3–107.0); p < 0.001. (B) Viral URTIs: median 148.0 ng/mL (IQR, 103.0–199.0) vs 70.8 ng/mL (IQR, 21.7–129.0); p < 0.001. (C) Viral LRTIs: median 61.5 ng/mL (IQR, 18.1–199.0) vs 22.6 ng/mL (IQR, 9.9–58.1); p = 0.011. Boxes indicate median and interquartile range, with whiskers indicate 1.5 times of IQR, and dots marking outliers beyond the whiskers.

MxA=Myxovirus resistance protein A. IQR= interquartile range. URTI=upper respiratory tract infections. LRTI=lower respiratory tract infections.

**Supplemental Figure 7.** Serial measurements and temporal profiles of MxA levels and body temperature in nine viral LRTI patients who had fever within 48 hours of the first MxA testing.


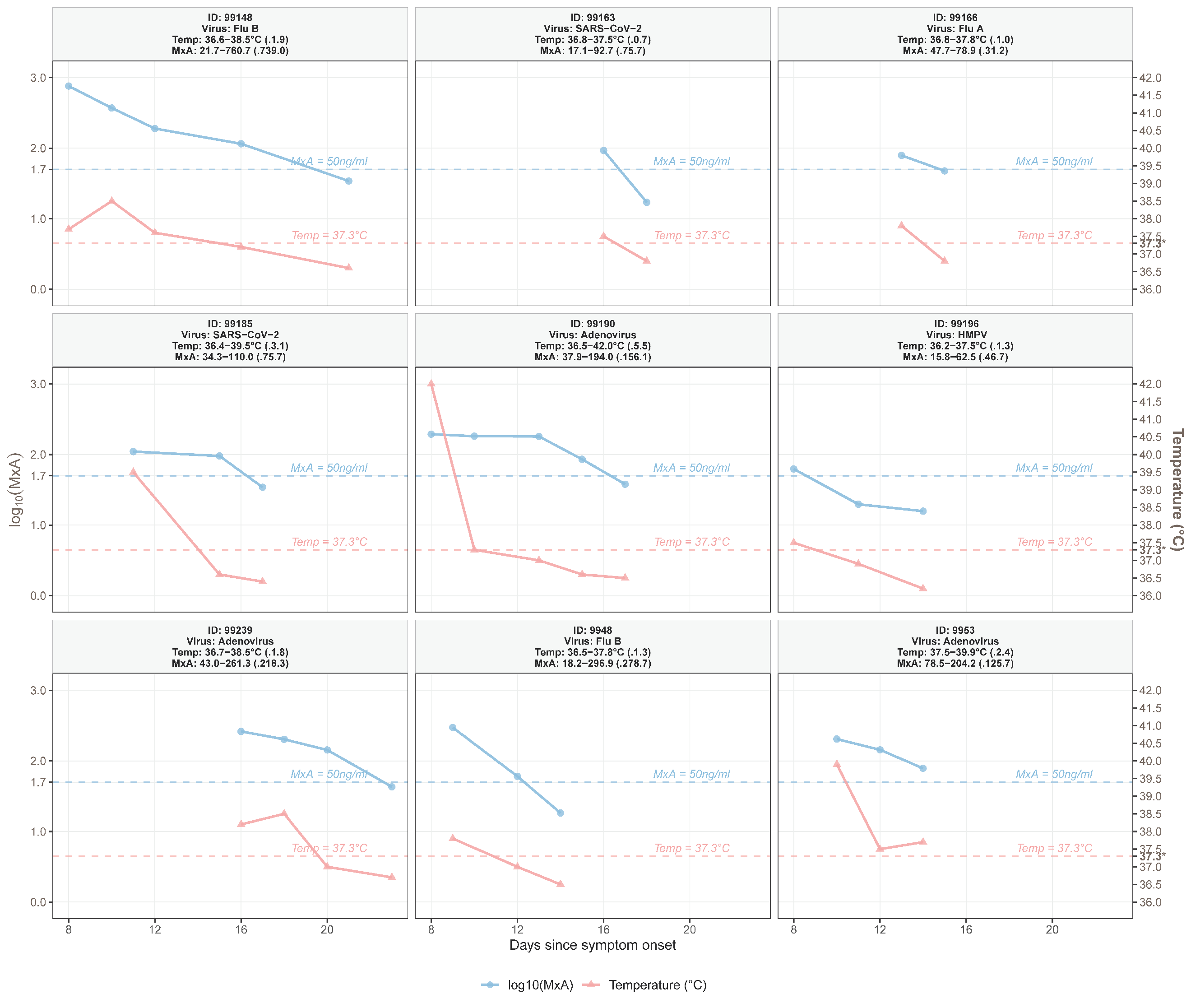


MxA=Myxovirus resistance protein A. LRTI=lower respiratory tract infections. Flu B=influenza B. Flu A=influenza A. HMPV= Human metapneumovirus.

**Supplemental Figure 8.** ROC curve of MxA to discriminate between viral and bacterial/fungal URTI requiring clinical intervention.


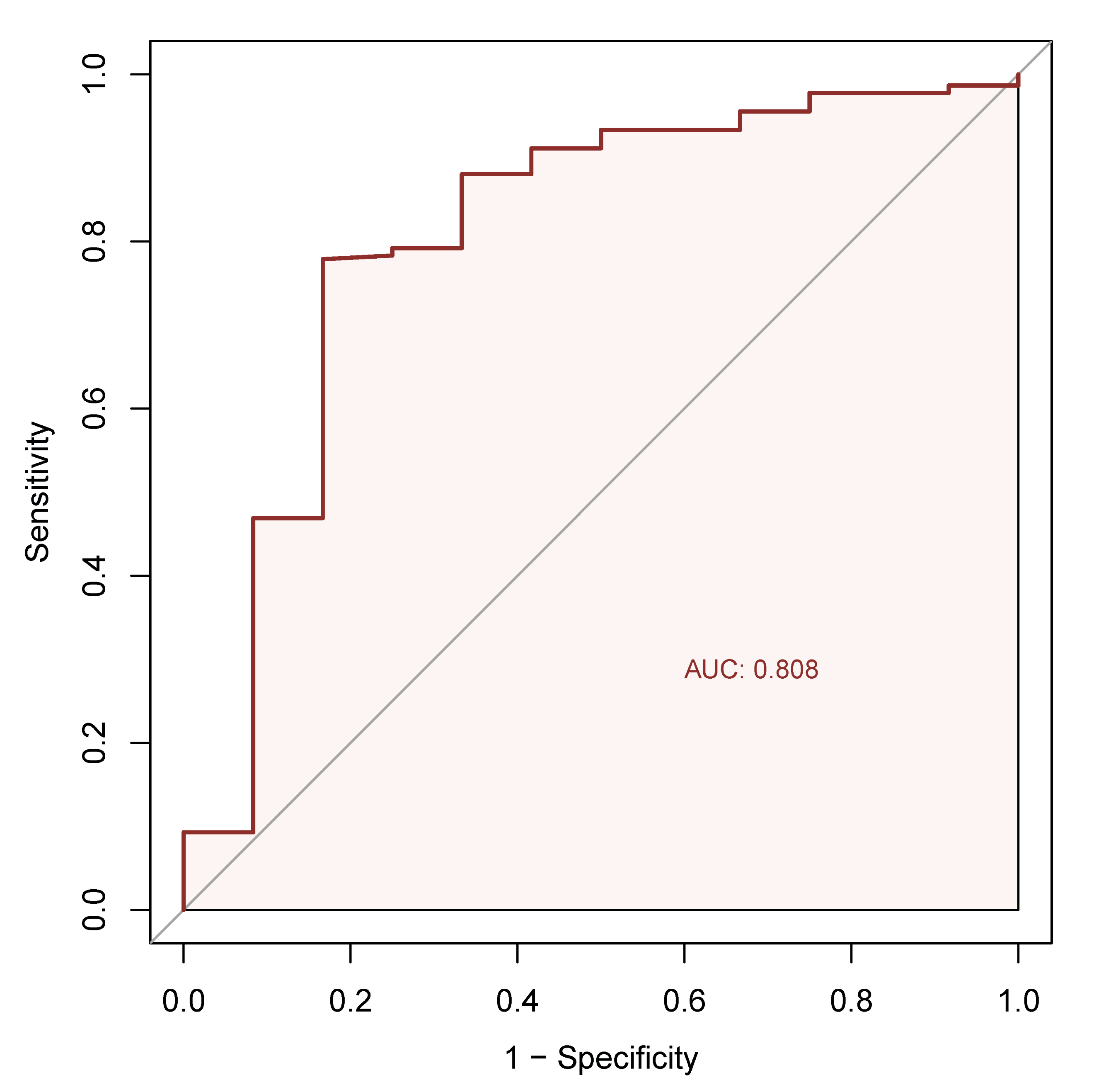


This figure shows the ROC curve of MxA for distinguishing viral from bacterial/fungal URTI, excluding typical bacteria-detected patients with WBC < 10×10⁹/L.

ROC=receiver operating characteristic. MxA=Myxovirus resistance protein A. URTI=upper respiratory tract infection.

**Supplemental Figure 9.** Boxplots of MxA levels in ARI patients and asymptomatic controls in sensitivity analyses based on clinically informed etiological classification


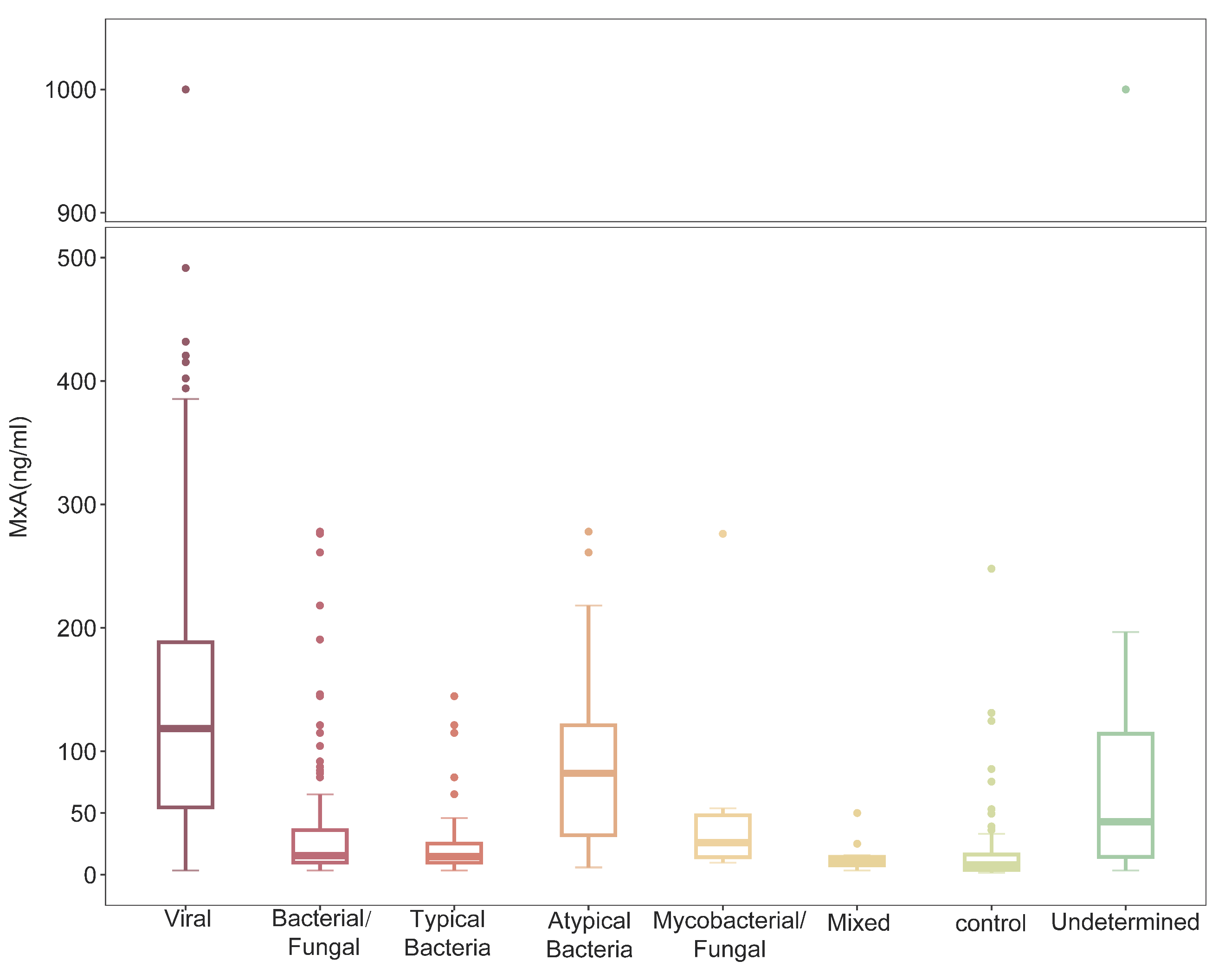


Boxplots of MxA levels in ARI patients and asymptomatic controls, categorized by pathogen group in a sensitivity analysis based on the clinically informed etiological classification. Boxes indicate median and interquartile range, with whiskers indicate 1.5 times of IQR, and dots marking outliers beyond the whiskers.

MxA=Myxovirus resistance protein A. ARI=acute respiratory infection.

**Supplemental Figure 10.** IPTW-weighted ROC curve evaluating the diagnostic performance of MxA for discriminating respiratory viral from bacterial/fungal infections.


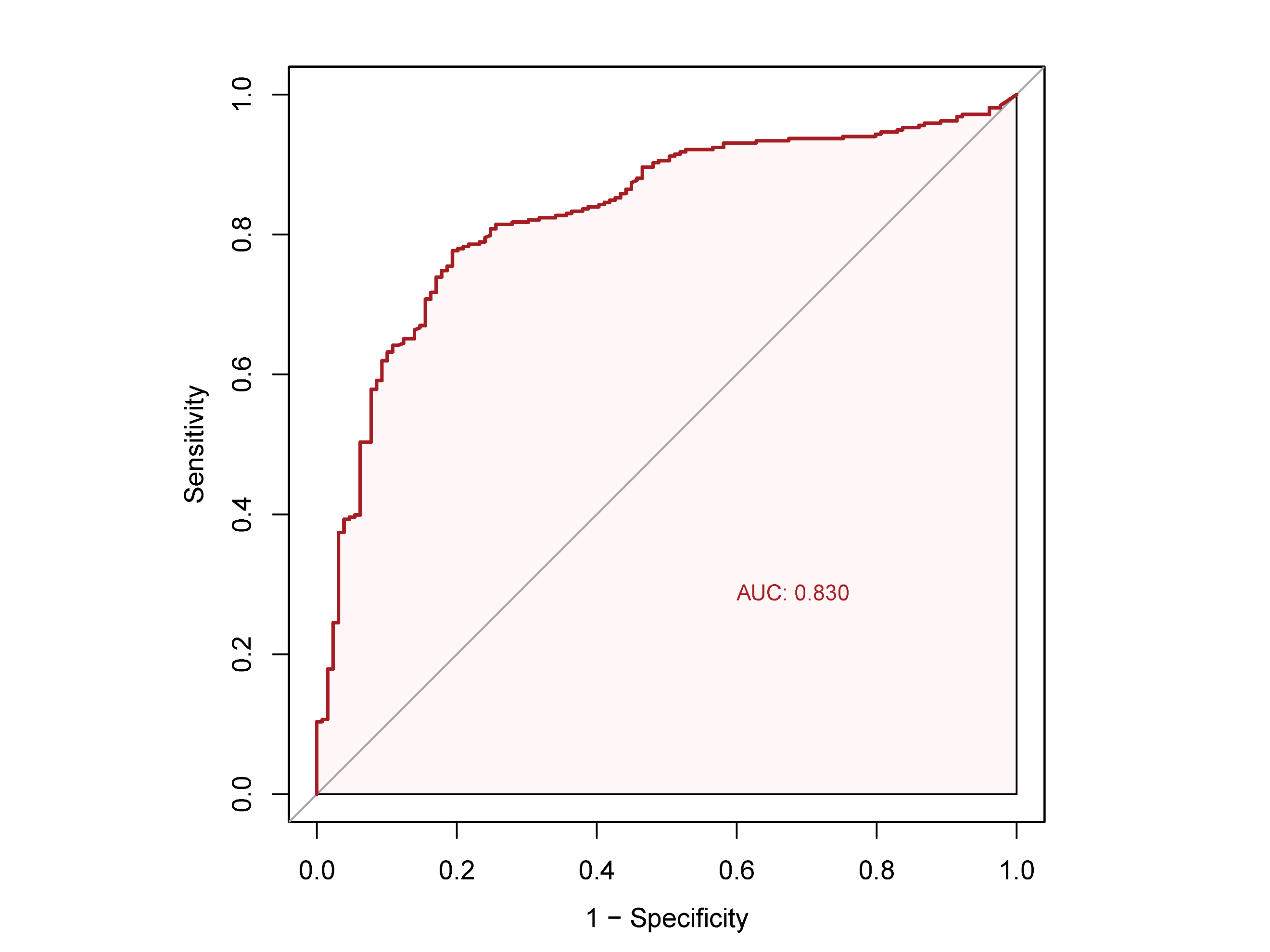


This figure shows the IPTW-weighted ROC curve of MxA for distinguishing respiratory viral from bacterial/fungal infections. Propensity scores were estimated using a logistic regression model including age, sex, duration of symptoms, comorbidities, immune status, infection site, and fever within 48 hours of blood sampling.

IPTW=inverse probability of treatment weighting. ROC=receiver operating characteristic. MxA=Myxovirus resistance protein A.

**Supplemental Figure 11.** Stacked bar chart of respiratory support categories across MxA levels in patients with viral LRTI.


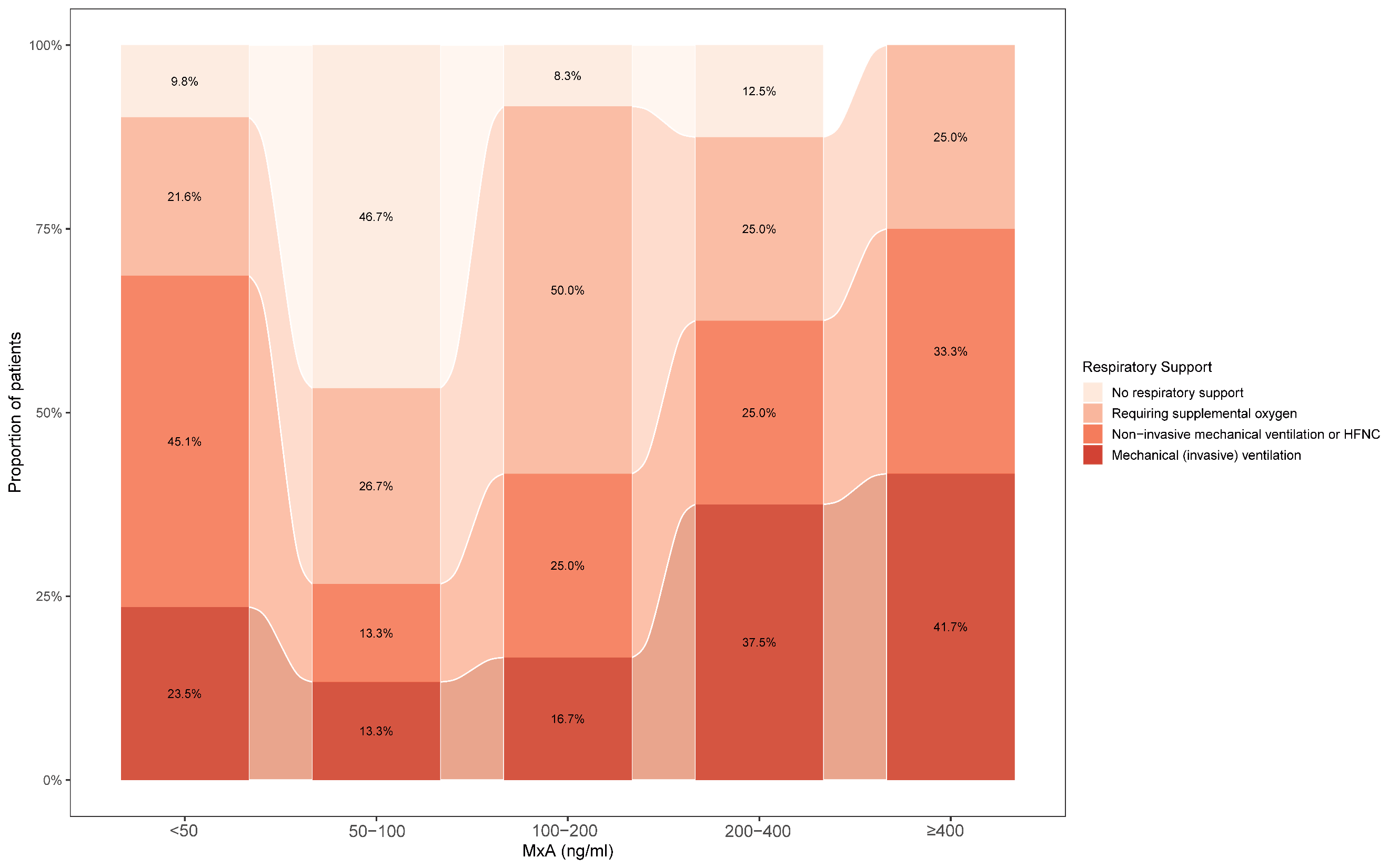


Stacked bar chart stratified by MxA levels, showing the proportions of patients receiving different types of respiratory support across MxA categories.

**Supplemental Figure 12.** ROC curves of CRP and CRP–MxA models for discriminating viral from bacterial/fungal infections.


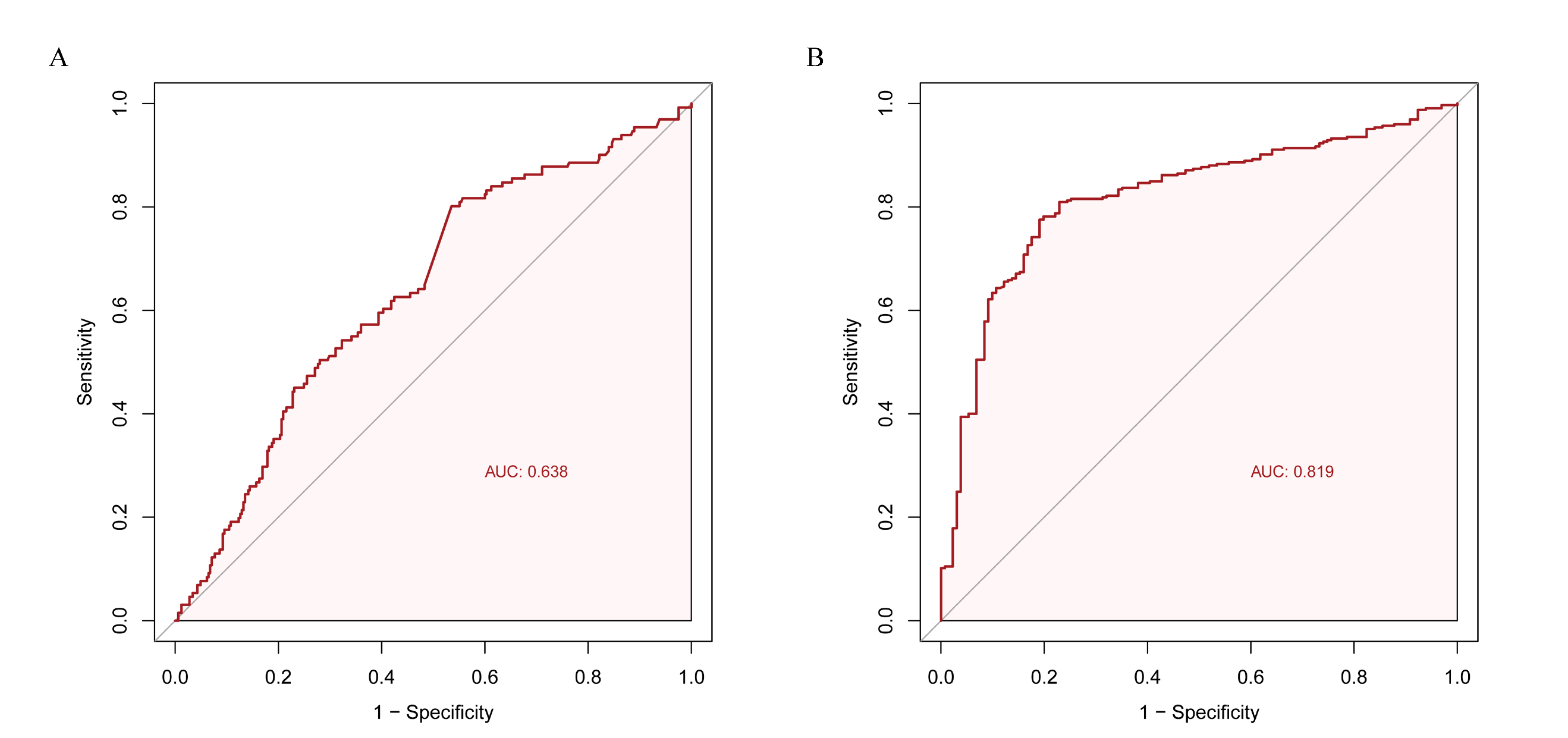


This figure shows (A) the ROC curve of CRP alone and (B) the ROC curve of the combined CRP–MxA model for distinguishing respiratory viral from bacterial/fungal infections.

ROC=receiver operating characteristic. CRP= C-reactive protein. MxA=Myxovirus resistance protein A.
