# Supplementary material for: Myxovirus resistance protein A to differentiate between viral and non-viral respiratory infections in adults: a prospective study
Source: Emerg Microbes Infect. 2026 Jan 16;15(1):2614734. doi: 10.1080/22221751.2026.2614734 (PMC12818327; doi:10.1080/22221751.2026.2614734)
Supplement: Appendix3.docx [file TEMI_A_2614734_SM3360.docx]

**Appendix 3. MxA Quantitative Measurement assay.**

Whole blood samples were collected for MxA quantitative measurement within 4 hours of sampling using a fluorescence immunochromatography (Zybio Inc., Chongqing, China) MxA detection kit and the Q20 automatic fluorescence immunoassay analyzer (Zybio Inc., Chongqing, China).

The MxA fluorescence immunochromatography detection strip consists of a sample pad, a conjugate pad, a nitrocellulose membrane, an absorbent pad, and a PVC backing plate. The conjugate pad is pre-coated with fluorescent microsphere-labeled anti-human MxA mouse monoclonal IgG1 (clone 22D4), as well as a fluorescent microsphere-labeled dinitrophenol (DNP)-bovine serum albumin (BSA). The test line on the nitrocellulose membrane is coated with anti-human MxA mouse monoclonal IgG1 (clones 27H9 and 19A8), while the control line is coated with anti-DNP antibodies.

Following the standard operating procedure, the whole blood sample is placed on the sample rack of the Q20 analyzer, which automatically aspirates 5 μl of whole blood and mixes it with the MxA sample diluent (Zybio Inc., Chongqing, China). A 70 μl aliquot of the mixture is then added to the sample pad of the test strip. The mixture migrates along the strip, where MxA in the mixture binds to the fluorescently labeled antibodies on the conjugate pad and is subsequently captured by the immobilized antibodies on the test line, generating a fluorescent signal proportional to the MxA level. Regardless of the MxA levels, the anti-DNP antibodies on the control line bind to the fluorescently labeled DNP-BSA complex, generating a constant fluorescent signal.

The Q20 analyzer automatically measures the fluorescence intensities of the test line and control line and calculates the MxA level based on a manufacturer-predefined internal standard curve. The total testing time is 8 minutes.
